# Three Extraction Methods in Combination with GC×GC-TOFMS for the Detailed Investigation of Volatiles in Chinese Herbaceous Aroma-Type Baijiu

**DOI:** 10.3390/molecules25194429

**Published:** 2020-09-27

**Authors:** Lulu Wang, Mengxin Gao, Zhipeng Liu, Shuang Chen, Yan Xu

**Affiliations:** State Key Laboratory of Food Science & Technology, Key Laboratory of Industrial Biotechnology of Ministry of Education & School of Biotechnology, Jiangnan University, Wuxi 214122, China; 15761632279@163.com (L.W.); mxgao97@163.com (M.G.); lzp940918@163.com (Z.L.)

**Keywords:** GC×GC-TOFMS, HS-SPME, SPE, SBSE, Chinese herbaceous aroma-type Baijiu

## Abstract

In this study, the detailed volatile compositions of Chinese herbaceous aroma-type Baijiu (HAB) were characterized by comprehensive two-dimensional gas chromatography-time of flight mass spectrometry (GC×GC-TOFMS). A total of 606 compounds were tentatively identified by similarity, mass spectral data, and retention indices, among which 247 compounds were positively verified by authentic standards. Esters were present in higher numbers (179), followed by aldehydes and ketones (111), and alcohols (81). In addition, there were also many terpenes (82), sulfides (37), furans (29), nitrogenous compounds (29), lactones (17), and so on. Meanwhile, the extraction effects of volatile components from different sample pretreatment methods (headspace solid-phase microextraction (HS-SPME), solid phase extraction (SPE), and stir bar sorptive extraction (SBSE)) for HAB were also revealed. The results indicated that HS-SPME has a better extraction effect on easily volatile compounds, such as alcohols and sulfides, especially for terpenes. SPE was particularly beneficial for the analysis of nitrogen-containing compounds; SBSE showed medium extraction ability for most types of compounds and was more suitable for the target analysis of trace content substances.

## 1. Introduction

As a traditional indigenous spirit and the most distilled liquor produced globally [1], Baijiu plays an important role in the Chinese traditional food industry, with nearly 8 million kiloliters of production in 2019 [2]. Baijiu is made from sorghum as the main raw material, produced by a solid-state spontaneous fermentation process, which accumulates a complex community of microorganisms contributing to the generation of complex layers of flavor [3,4]. Due to the differences in production technology and aroma characteristics, Baijiu can be divided into different aroma-type categories, including soy sauce aroma type, light aroma-type, strong aroma-type, and herbaceous aroma-type Baijiu (HAB), etc. Among them, HAB is produced from sorghum mixed with more than 100 Chinese herbs [5], which gives the distillate a distinctive flavor and creates more aroma active substances.

Volatile compositions are often regarded as the main characteristics to determine Baijiu quality and are essential for consumers’ criteria; therefore, most studies focus on the identification of volatile components in Baijiu. Gas chromatography-flame ionization detection (GC-FID), gas chromatography-mass spectrometry (GC-MS), and other techniques have been gradually applied to the study of Baijiu, and hundreds of volatile components have been identified so far. Nevertheless, because the resolution of one-dimensional gas chromatography (1DGC) has a limit to separating mixtures including hundreds or even thousands of components, it is often necessary to combine normal phase chromatography and other complex pre-separation methods to assist in the separation and identification of volatile components in Baijiu samples [6,7]. Therefore, to meet the requirement of stronger separation energy, comprehensive two-dimensional gas chromatography (GC×GC) has emerged. GC×GC involves the combination of two capillary columns with different separation mechanisms through a single modulator. With properly selected orthogonal separation mechanisms, GC×GC allows for the separation of a large number of compounds in a single chromatographic run due to the added selectivity of the second column and inherently high peak capacity [8]. GC×GC has been a powerful technique for analyzing volatile components in highly complex samples [9,10], such as petroleum [11], environmental samples [12], essential oils [13], and wines [14]. Through acquisition of a large amount of data from samples based on GC×GC, significantly different compounds from different regions or varieties were recognized by means of multivariate analysis [15,16,17]. GC×GC has also been used to create sample-specific fingerprints for sample differentiation [18]; for example, Cordero et al. used GC×GC for the creation of two-dimensional (2D) fingerprints for roasted hazelnuts from different cultivars, varieties, and geographical origins [19]. In addition, Gracka et al. used GC×GC to monitor the changes in volatile compounds related to roasting conditions [20].

Despite the significant benefits offered by GC×GC for the separation and identification of volatiles, sample preparation is also a critical step when characterizing the volatile compositions in such a complex matrix. Headspace solid-phase microextraction (HS-SPME) has been by far the most applied sample preparation method in GC×GC, followed by solid phase extraction (SPE), and stir bar sorptive extraction (SBSE) [21]. A series of validated HS-SPME methods have been proposed for targeted analyses of volatile compounds in a variety of samples [22,23], while research found that HS-SPME has limited application for some influential high-boiling compounds [8]. Accordingly, to compensate for these shortcomings, SPE combined with GC×GC was utilized for the detailed investigation of particularly low-level semi-volatiles and obtained a satisfactory result in wine [24].

In the recent years, GC×GC has been gradually applied to the component analysis of soy sauce aroma-type Baijiu [25] and strong aroma-type Baijiu [26], and more than 1000 volatile components have been identified. However, there are few studies of HAB [27,28], and no systematic analysis using GC×GC has been performed so far. Hence, we analyzed HAB by means of comprehensive two-dimensional gas chromatography-time of flight mass spectrometry (GC×GC-TOFMS) for the purpose of overall characterization of volatiles and revealed the volatile compound profile, and potentially key aromatic compounds. Meanwhile, three pretreatment methods (HS-SPME, SPE, and SBSE) in combination with GC×GC-TOFMS were used for the first time to compare the extraction ability on a complex Baijiu sample and determine the biased analysis of some methods for certain groups of compounds.

## 2. Results and Discussion

### 2.1. GC×GC-TOFMS Separation and Identification of Volatile Components in HAB

**Figure 1:**
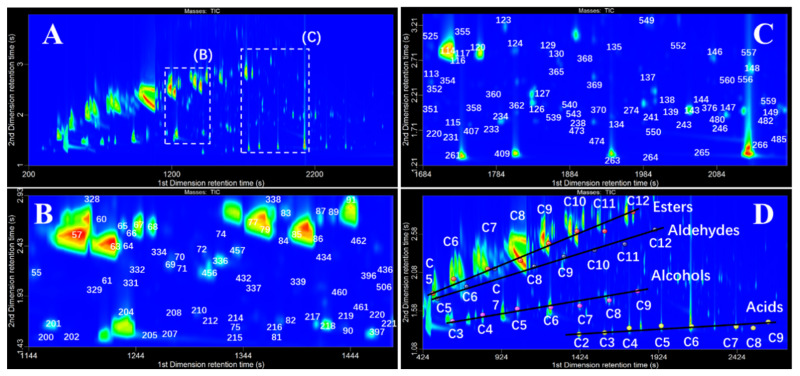
(**A**) Complete 2D contour plot; (**B**,**C**) present detailed portions of the contour plot to illustrate some of the identified compounds. Compound numbers correspond to Table S1. (**D**) GC × GC distribution of homologous series. Esters: ethyl propionate, ethyl butanoate, ethyl valerate, ethyl hexanoate, ethyl heptanoate, ethyl octanoate, ethyl nonanoate, and ethyl decanoate. Aldehydes: pentanal, hexanal, heptanal, octanal, nonanal, decanal, undecanal, and dodecanal. Alcohols: propanol, butanol, pentanol, hexanol, heptanol, octanol, and nonanol. Acids: acetic acid, propanoic acid, butanoic acid, pentanoic acid, hexanoic acid, octanoic acid, nonanoic acid, and decanoic acid.

**Figure 2:**
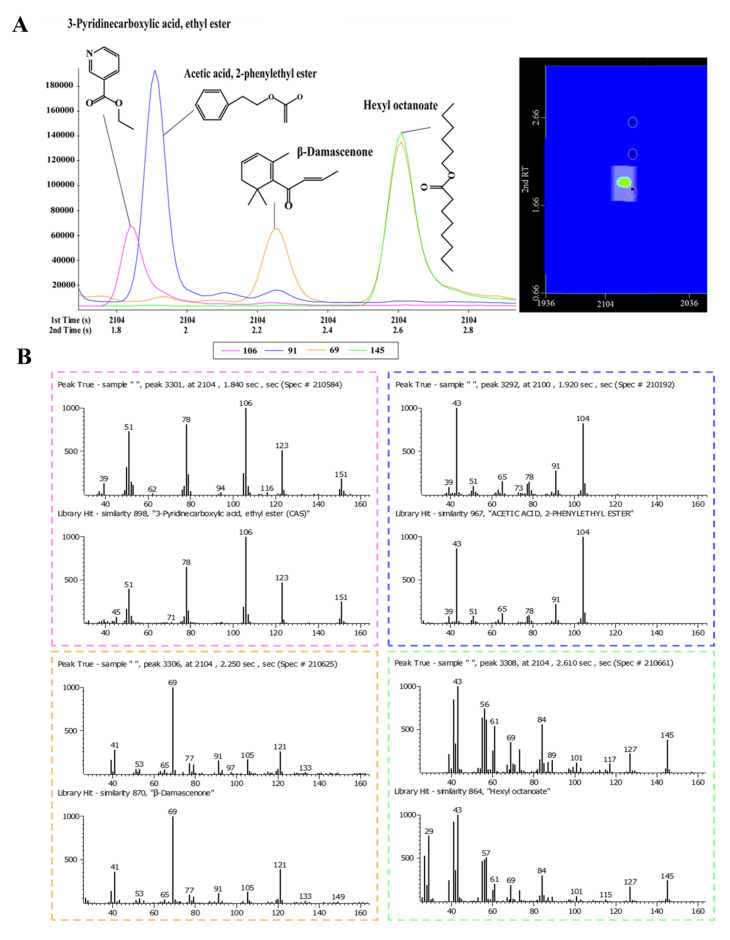
(**A**) Four peaks shown in the two-dimensional chromatogram and modulated peaks of four compounds found in Chinese herbaceous aroma-type Baijiu. (**B**) Deconvoluted mass spectra of compounds.

GC×GC-TOFMS was used for the overall characterization of volatile components in HAB in this study based on the higher capacity, significantly enhanced resolving power, and spectral deconvolution function. As Baijiu mainly consists of polar compounds, such as esters, alcohols, aldehydes, and acids, a (polar × medium-polar) column combination can be more beneficial to the separation [25]. Figure 1A is the 2D chromatogram obtained for HAB analyzed by HS-SPME combined with GC×GC-TOFMS. Ordered chromatograms of homologous series in HAB are observed using the above reversed-type column set. Baijiu has a complex matrix consisting of a large number of volatiles with wide-ranging physicochemical properties, and abundant coelution is observed on conventional 1DGC. This restriction is overcome in GC×GC by subjecting the sample to separation based on two different mechanisms, e.g., polarity in 1D and mid-polarity in 2D. Figure 1B,C illustrates the effectiveness of this approach for HAB analysis. Obviously, the number of compounds shown in this figure could not be separated using conventional 1DGC methods. In fact, only approximately 1/5 of the compounds detected here can be fully resolved in one dimension. As shown in Figure 2A, acetic acid 2-phenylethyl ester, 3-pyridinecarboxylic acid ethyl ester, hexyl octanoate, and β-damascenone were coeluted during 1DGC separation, and this overlapping peak makes qualitative and quantitative analysis difficult. Figure 2A shows that these four compounds are easily separated in the 2D plot. Analytes flowing from the 1st column were sequentially separated by 2nd columns with different retention mechanisms, and the interference of coeluted components was efficiently reduced. Figure 2B shows the mass spectra of the four compounds compared to the mass spectra in the NIST library, and the results indicate that identification of the compound is accurate.

Another advantage of GC×GC is the generation of structured chromatograms. The compounds with similar chemical structures will be grouped in a 2D plot. As shown in Figure 1D, the presence of four homologous series compounds was observed for some straight-chain esters, alcohols, acids, and aldehydes. The lines described in the graph show a tendency of organized distribution of these components in the 2D space, and labels represent the carbon atom number of the molecule. The organized distribution of homologous members can be predicted or confirmed, so it is very useful for reliable identification. Esters and alcohols have lower polarity, so they eluted early on the 1st column. Polar acid compounds were retained better in the 1D column, because of their strong polarity; they eluted at higher temperatures, so they eluted earlier on the second dimension and appeared at the bottom. However, apparent 2D tailing was observed for acid compounds, which may cause more coelutions and influence the accurate identification, especially of minor constituents. Tailing in the 2nd dimension was related to the incompatibility of the polar compounds with the mid-polar stationary phase used in 2D.

Nontargeted analysis is performed when it is desirable to have knowledge of all the components in a mixture. In this study, more than 3000 chromatographic peaks with signal-to-noise (S/N) ratios greater than 100 were recognized by deconvolution. Then, the deconvoluted mass spectra were compared with NIST 2014 and Wiley 9 spectral libraries using Chroma TOF4.61.1.0 software with a match value of 70% as the minimum requirement, and 1266 compounds were retained. Next, 472 unwanted search results were eliminated. Finally, a total of 606 compounds were verified by comparing retention indices and mass spectra to those of reference standards. Among them, 247 compounds were positively verified by authentic standards.

### 2.2. Comparison of Pretreatment Methods

**Figure 3:**
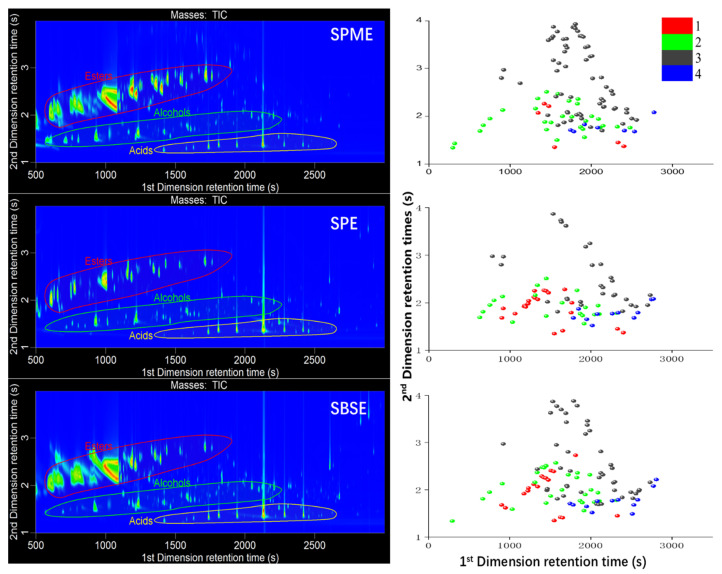
Total ion chromatogram (TIC) contour plot obtained from the HS-SPME-GC×GC-TOFMS, SPE-GC×GC-TOFMS, and SBSE-GC×GC-TOFMS analysis of herbaceous aroma-type Baijiu, and 4 classes of compounds distributed in contour plot (red balls are nitrogenous compounds, green balls are sulfides, gray balls are terpenes, and blue balls are lactone compounds).

**Figure 4:**
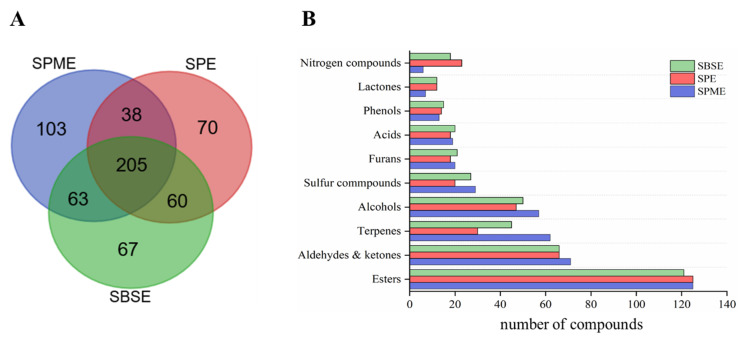
Comparison of identification compounds obtained by HS-SPME-GC×GC-TOFMS, SPE-GC×GC-TOFMS, and SBSE-GC×GC-TOFMS. (**A**) (Venn diagram) and (**B**) (Bar plot graph displaying compound distribution according to chemical class).

Figure 3 shows the 2D plot of HAB extracts obtained by three different pretreatment methods (HS-SPME, SPE, and SBSE). Table S1 lists the 606 compounds identified in this study, grouped according to different chemical classes. This result showed the most detailed characterization of volatile constituents in HAB for the first time. Figure 4 presents the correlation of HAB analytes identified by three pretreatment methods, and only 205 compounds were commonly identified, which shows the great difference among the three pretreatment methods. Table 1 compares the number of compounds identified in each class using HS-SPME, SPE, and SBSE.

HS-SPME is based on the establishment of partition equilibrium of the analytes between the polymeric stationary phase, which covers a fused silica fiber, and the matrix of the sample. It is a simple, rapid, and inexpensive technique in which the extraction and concentration processes are performed simultaneously; furthermore, only small sample volumes are required [29]. A total of 409 volatile compounds were identified by HS-SPME-GC×GC-TOFMS, including esters, alcohols, sulfides, and terpenes; however, lactones and nitrogenous compounds were poorly detected. The results indicated that HS-SPME was particularly beneficial for the analysis of volatile compounds, but defective for the extraction of some high-boiling volatile compounds.

SPE has also been reported to have the functions of extraction, enrichment, and rinsing. Abundant adsorbent material can provide high extraction capacity when properly optimized [21]. SPE is supposed to be the complementary nature of the extraction techniques for HS-SPME, which is best exploited for the analysis of semi-volatiles. Several peaks present at the end of the contour plot by SPE were not detected when using HS-SPME, such as *γ*-dodecalactone, ethyl *cis*-9-octadecenoate, ethyl linoleate, and ethyl vanillate. In addition, more nitrogenous compounds were detected when using SPE protocol. However, some volatile compounds may be lost, including terpenes and volatile sulfides, because the Baijiu sample was exposed to atmosphere during extraction.

SBSE was initially introduced in 1999 as a miniaturized and solvent-free extraction technique for aqueous samples. Compared with HS-SPME, SBSE provides greater analytical sensitivity and reaches much lower detection and quantification limits. The reason is that the enrichment factor for SBSE is higher than that of HS-SPME using the same stationary phase, because of the 50–250 times larger volume of extraction phase on the stir bar [30]. However, large amounts of stationary phase extracted excessive amounts of solute, thus overloading the GC×GC system (particularly the 2nd column). After split injection (20:1), more terpenes and sulfides were obtained than by the SPE method, due to the apolar adsorbent material PDMS. On the other hand, more high-boiling compounds, including higher fatty acid esters, acids, nitrogenous compounds, and lactones, were obtained.

### 2.3. Volatile Components in HAB

A total of 606 volatile compounds were identified in this study, among which esters were present in the highest number (179), followed by aldehydes and ketones (111), terpenes (82), alcohols (81), sulfides (37), furans (29), nitrogenous compounds (29), acids (23), phenols (18), and lactones (17).

#### 2.3.1. Skeleton Components

**Figure 5:**
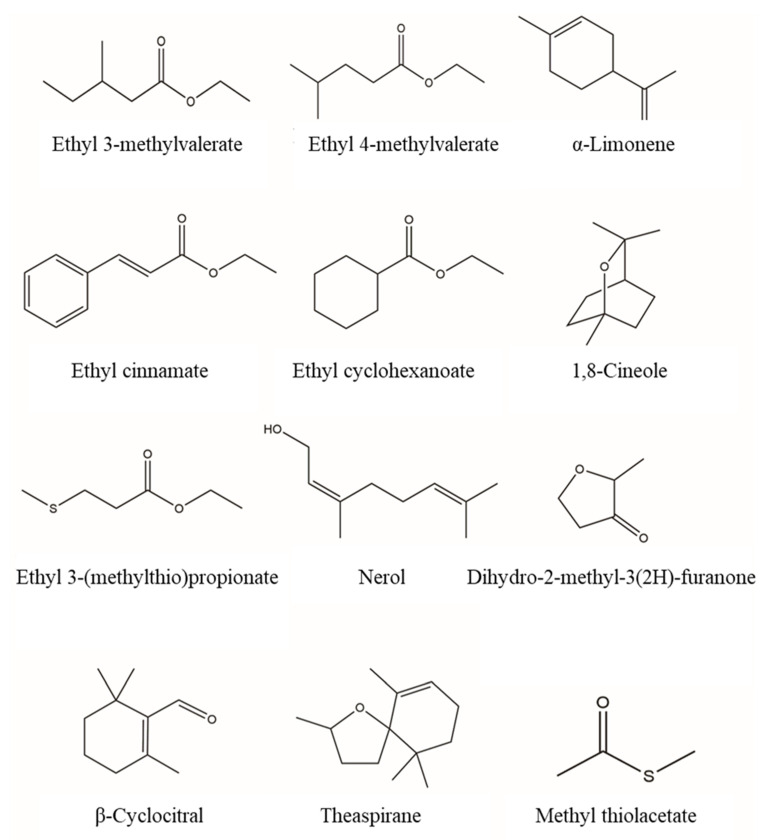
Chemical structure of some aroma compounds first reported in Chinese herbaceous aroma-type Baijiu.

Esters, alcohols, acids, aldehydes, and ketones are the skeletal components of Chinese Baijiu. A total of 179 esters were detected in this study. Among them, ethyl esters were the most representative, and the ethyl esters homologous C2–C12 and C14–C18 were all detected. Ethyl acetate, ethyl butanoate, and ethyl hexanoate are the key aroma compounds in Baijiu, which mainly contribute to its fruity and sweet aroma. In addition, some compounds with very low odor thresholds that cannot be found in the previous literature also contribute to the overall aroma, for example (Figure 5), ethyl 3-methylvalerate has an odor threshold of 8 ng/L and contributes to a strawberry flavor [31]; ethyl cyclohexanoate has an odor threshold of 1 ng/L and reveals strawberry and anise aroma [32]; ethyl cinnamate is known for its honey and cinnamon flavor with an odor threshold of 1 μg/L [33].

Aldehydes and ketones were the second major categories of identified volatiles, amounting to a total of 111 compounds, and the homologous series of straight-chain aldehydes from C2–C12 were all detected. (*E, Z*)-2,6-nonadienal is the strongest aroma aldehyde compound in HAB and is described as having a strong cucumber aroma [27]; 3-methyl-butanal presents cocoa and almond aroma with an odor threshold of 0.5 μg/L [27]; 2,3-butanedione has been described as contributing a butter aroma with an odor threshold of 100 μg/L [33]; (*E*)-2-octenal is a key aroma odorant of Chinese chixiang aroma-type Baijiu, which has a fatty flavor with an odor threshold of 15.1 μg/L [27]. 3-Methyl-butanal, 2,3-butanedione and (*E*)-2-octenal were identified in this study for the first time.

Alcohols are highly volatile constituents of alcoholic beverages transformed from sugar during fermentation. Among them, 39 out of the 81 alcohols were confirmed using authentic standards, and the homologous series of saturated straight-chain primary alcohols from C3-C12 were all detected. 2-Butanol, 1-butanol, and 3-methyl-1-butanol have odor thresholds of 50, 2.73, and 179 mg/L, respectively, in Baijiu. They are also important aroma compounds in HAB, contributing to the fruit or mellow flavor [27].

A total of 23 acid compounds were identified in this study, most of which are saturated monocarboxylic fatty acids. The homologous series of straight-chain monocarboxylic fatty acids from C1–C10, C14, and C16 were all detected, among which butanoic acid, pentanoic acid, and hexanoic acid play an important role in the flavor of Baijiu, which contributes to rancid and cheesy odors [27]; 2-methyl butyric acid and phenylacetic acid have relatively low odor thresholds of 5.9 and 1.4 mg/L, respectively, and are the key food odorants (KFO) [34].

#### 2.3.2. Terpenes

Many terpenoids have important physiological activities, although they exist with low contents in Baijiu. A total of 82 terpenoid compounds (Table 2) were detected in this study, including mono- and polyterpene hydrocarbons, alcohols, carbonyls, and esters. This represents a significant improvement in the number of terpenoids detected by GC×GC-TOFMS compared to a previous report (only 41 compounds) [28]. Terpenes are well-known varietal compounds of Vitis vinifera, and raw materials are important sources of terpenoids; furthermore, most terpenes exist in grapes, and their contribution to wine aroma is significant [35]. During the production process of HAB, more than 100 Chinese herbs are mixed in the raw materials, which create more terpenoid compounds and give Baijiu a distinctive flavor. In addition, substantial evidence also exists to show the formation of terpene-related compounds during fermentation and aging.

Linalool, geraniol, and β-citronellol are common terpenes in Baijiu and present floral aroma properties. The odor perception thresholds of these compounds are 13.1 μg/L, 120 μg/L, and 300 μg/L [36], respectively. Geosmin is known for its beet, earth aroma as an off-flavor compound, whose odor threshold is 0.1 μg/L in 46%vol ethanol aqueous solution [37]. β-Ionone is believed to be responsible for the characteristic violet and floral aroma, whose concentration in Baijiu is generally 0.3–2.2 μg/L, and the odor threshold in 46%vol ethanol aqueous solution is 1.3 μg/L [36]. In addition, a series of terpenes with low thresholds were also found in this study for the first time (as shown in Figure 5); for example, 1,8-cineole presents a fresh odor with an odor threshold of 0.26 μg/L, and β-cyclocitral has an odor threshold of 5 μg/L [38].

#### 2.3.3. Sulfides

Volatile sulfides play a remarkable role in the aroma of food and beverages, even when present at low concentrations [39,40]. Recently, Wang et al. found that the imbalance of sulfides will lead to the off-odor in soy sauce aroma-type Baijiu; however, these compounds might contribute to the overall aroma of Baijiu at relatively low concentrations [41]. In this study, a total of 37 sulfides (Table 3) were identified, of which 28 compounds have not been reported before in Baijiu. Sulfides usually have a relatively low odor threshold and contribute to the onion, cabbage, and sulfur aroma. Dimethyl trisulfide is an important sulfide in Baijiu, and it is well known for the onion and cabbage aroma with an odor threshold of 0.36 μg/L [42]. Methional is characterized by cooked potatoes and has a perception threshold of 7.1 μg/L [42]. Several sulfides were first reported in HAB, and s-methyl ester butanethioic acid shows a sulfurous and cheesy aroma [43], 5-Methyl-2-formylthiophene is described as moldy odor, and 1,2,4-trithiolane contributes the roasted beef and sulfurous aroma.

#### 2.3.4. Cyclic Components

Furans, phenols, and lactones were classified under this group. In this research, a total of 29 furans were detected. Compounds of 1-(2-furanyl)-ethanone, 5-methyl-furfural, 2-acetyl-5-methylfuran, and 2-furanmethanol have been described as contributing a honey, caramel odor [27]. Dihydro-2-methyl-3(2*H*)-furanone reveals sweet, bread and buttery aroma with a sensory threshold of 5 ng/L [44]. This kind of compound mainly contributes to the sweet aroma of Baijiu.

Eight out of the 18 phenols are first detected in HAB. 2-Methoxy-4-ethylphenol, 2-methoxy-4-methylphenol and 2-methoxy phenol were reported to be important aroma compounds in HAB, revealing smoke, sweet, and spice aroma with odor thresholds of 123 μg/L, 315 μg/L, and 13 μg/L, respectively. 2-Methyl-phenol and 3-ethyl-phenol are key food odorants [34], and they have not been reported in HAB before.

A total of 17 lactone compounds were detected in the current study, of which 12 were identified in Baijiu for the first time. γ-Caprolactone and γ-nonalactone present weak sweet, fruity aroma in HAB [27]. γ-Decalactone has been reported to contribute to peachy and fatty aroma with a threshold of 5000 μg/L [33]. γ-Dodecalactone is responsible for the sweet and floral aroma with a threshold of 7 μg/L [33]. γ-6-(*Z*)-dodecenolactone was first detected in Baijiu and is associated with sweet and fruity aroma, and its odor threshold is 700 ng/L [45].

#### 2.3.5. Nitrogenous Components

In this study, a total of 29 nitrogenous compounds were detected in HAB, mainly consisting of pyrazines, pyrroles, and pyridines. Tetramethyl-pyrazine was previously described as a baked flavor in HAB [27]. Isopropylpyrazine was identified in Baijiu for the first time.

## 3. Materials and Methods

### 3.1. Reagents and Chemicals

A commercially available Dongjiu Baijiu (54% ethanol) was used, which was produced by Guizhou Dongjiu Co., Ltd. (Guizhou, China) according to the National Standard of Herbaceous Aroma-Type Baijiu (DB52/T550). All chemical standards with high-purity grade (GC grade) and C7-C30 n-alkane mixture were obtained from Sigma-Aldrich Co., Ltd. (Shanghai, China). Organic solvents of methanol (HPLC grade), ethanol (HPLC grade), and dichloromethane (HPLC grade) were purchased from J&K Scientific Co., Ltd. (Beijing, China). Sodium chloride (NaCl) and anhydrous sodium sulfate (Na_2_SO4) were purchased from China National Pharmaceutical Group Corp. Ultrapure water was obtained from a Milli-Q purification system (Millipore, Bedford, MA, USA).

### 3.2. Sample Extraction Methods

#### 3.2.1. HS-SPME

An automatic headspace sampling system (MultiPurpose Sample MPS 2 with a SPME adaptor, from Gerstel Inc., Muülheim, Ruhr, Germany) with a 50/30 μm divinylbenzene/carboxen/polydimethylsiloxane (DVB/CAR/PDMS) fiber (2 cm, Supelco Inc., Bellefonte, PA, USA) was used to extract the volatile compounds. Following a method presented in the literature [41], the Baijiu sample was diluted with ultrapure water to a final concentration of 10% ethanol by volume. A total of 10 mL diluted Baijiu sample was transferred into a 20 mL screw-capped vial and saturated with 3 g of NaCl. Then, the sample was equilibrated at 40 °C for 5 min and extracted for 40 min at the same temperature under stirring at a rotation speed of 250 rpm. The extracts were desorbed in a GC splitless injector port at 250 °C for 5 min.

#### 3.2.2. SPE

SPE was based on a slightly modified method described by Chen et al. [46]. A total of 10 mL HAB was diluted with ultrapure water to 50 mL and saturated with 15 g of NaCl. The SPE cartridge (0.8 cm internal diameter, 12 mL internal volume, Sigma Aldrich, Shanghai, China) was consecutively conditioned using 20 mL of dichloromethane, 20 mL of methanol, and 30 mL of ultrapure water. A total of 50 mL diluted sample was passed through the Lichrolut EN cartridge at a flow rate of 2 mL/min. After the sample had been loaded, 30 mL ultrapure water was used to rinse the sorbent. Then, the sorbent was dried by letting the air pass through it (−0.6 bar, 10 min). Extracts were recovered by elution with 10 mL of dichloromethane, concentrated under a gentle stream of nitrogen to a final volume of 500 μL, and stored at −20 °C until analysis. Finally, 1 μL extracts were injected into the GC splitless injector port at 250 °C with 450 s acquisition delay.

#### 3.2.3. SBSE

In this study, SBSE was carried out according to the description in the literature [47]. Stir bars (Twister) coated with PDMS (10 mm length × 1.0 mm thickness) were obtained from GERSTEL. Prior to use, the stir bar was conditioned for 30 min at 280 °C in a flow of helium. The Baijiu sample was diluted with ultrapure water to a final concentration of 10% ethanol by volume. A 10 mL diluted sample was saturated with 3 g NaCl in a 20 mL glass vial, and a stir bar was immersed in the sample for enriching the substance. Then, the sample was placed in an agitation plate at 25 °C and extracted at 800 rpm for 90 min. After extraction, the stir bar was removed with a magnetic rod (twister taking tool) and forceps, rinsed briefly with ultrapure water to remove ethanol, and dried with lint-free tissue, followed by placement in a sample holder for GC×GC-TOFMS analysis.

An automatic headspace sampling system was used to analyze the extracts in this study. The stir bar was placed in a glass thermal desorption liner and thermally desorbed by programming the twister desorption unit (TDU) from 35 °C heated at a rate of 700 °C/min to a final temperature of 270 °C and held for 5 min. TDU injection was in split ratio of 20:1 mode during thermal desorption. A cooled injection system (CIS4) was used in the GC×GC-TOFMS system. CIS4 was in solvent vent mode with a venting flow of 60 mL/min for 4.7 min at a venting pressure of 80 kPa. The initial temperature of CIS4 was kept at −60 °C for 0.2 min, ramped at a rate of 10 °C/s to a final temperature of 250 °C, and held for 3 min.

### 3.3. GC×GC-TOFMS Instrumentation

Experiments were performed on a LECO Pegasus^®^ 4D GC×GC-TOFMS system (LECO Corp., St. Joseph, MI, USA). This instrument consisted of an Agilent 7890B GC (Agilent Technologies, Palo Alto, CA, USA) incorporating LECO’s thermal modulator (dual-stage quad-jet) and a secondary oven mounted inside the primary GC oven. The column set consisted of a 60 m × 0.25 mm × 0.25 μm DB-FFAP (Agilent Technologies, Palo Alto, CA, USA) primary column and a 1.5 m × 0.25 mm × 0.25 μm Rxi-17Sil MS secondary column (Restek, Bellefonte, PA, USA). Ultrahigh purity helium was used as the carrier gas at a constant flow of 1.00 mL/min. The separation was performed using the following temperature program: 45 °C kept for 3 min, ramped at 4 °C/min to 150 °C and held for 2 min; reaching 200 °C at 6 °C/min and 230 °C at 10 °C/min for 20 min. The secondary oven was operated at 5 °C higher than the primary oven throughout. The modulator was offset by +20 °C in relation to the primary oven. A modulation period of 4 s (hot pulse of 0.80 s) was used.

The TOFMS parameters included ion source of 230 °C and transfer line of 240 °C, electron energy of -70 volts, acquisition of 1430, mass range of 35–400 amu, and acquisition rate of 100 spectra/s.

### 3.4. Data Processing

ChromaTOF version 4.61.1.0 software (LECO Corp., St. Joseph, MI, USA) was used for peak finding, mass spectral deconvolution, peak area integration, and library searching. Automated peak finding and spectral deconvolution with a baseline offset of 0.5 and S/N of 100 after evaluating serval options (i.e., 25, 50, 100, 150, and 200). All analyses were performed in triplicate for each extraction method. The existence of the compound is considered reliable only when the number of detections is greater than 2 at the same retention time. Tentative identification was based on the comparison of mass spectra with the NIST 2014 and Weliy 9 databases using a minimum similarity value of 700 as the criterion, as well as experimentally determined linear retention indices compared to NIST library values. A series of n-alkanes were analyzed under the same conditions to determine first dimension linear retention indices (LRIs) for each compound. A maximum deviation of 30 between the experimental and literature RI values was used as the criterion. Some identification results may be consistent with MS and RI identification, but the 2nd dimensional retention time may not meet the linear distributions of homologous series. The ordered chromatograms of homologous series can also be used for identification. In addition, positive verification of 247 compounds (~41% of the total number) was based on comparison of retention time with authentic standards.

## 4. Conclusions

The combination of HS-SPME, SPE, and SBSE sample preparation methods coupled with GC×GC-TOFMS analysis enabled us to (tentatively and positively) identify 606 compounds in HAB. Many low content compounds that have never been reported before were identified for the first time. Especially for terpenes, 41 more compounds were identified than previously reported, which are important physiologically active substances in HAB. Furthermore, the contributions of some important compounds were studied in terms of aroma characteristics and odor thresholds. Meanwhile, the three extraction methods show distinct differences and biases for specific analytes. HS-SPME preferred the analysis of alcohols, sulfur-containing, and terpenes compounds; SPE generally revealed more high-boiling compounds, such as lactones and nitrogenous compounds; SBSE showed general extraction ability for all types of compounds, but too much adsorption led to chromatogram overload, making it suitable for the identification of trace content substances. Therefore, the analysis of volatiles in such a complex sample requires multiple pretreatment methods coupled with GC×GC-TOFMS. Importantly, the method can be applied to other alcoholic beverage systems for the determination of the specific kinds of volatile compounds. This approach proved beneficial for the analysis of terpenes, lactones, and sulfur containing compounds, which are important flavor contributors of Baijiu. In addition, the development of this technique laid a foundation for the quantitative determination of the content substances at very low levels (in the region of μg L^−1^ and lower). Based on the feasibility of accurate quantification, it is hoped that this method can be used to monitor the formation of key aroma substances in the production process of Baijiu.

## Figures and Tables

**Table 1:** Comparison of volatile compounds detected in Chinese herbaceous aroma-type Baijiu using HS-SPME-GC×GC-TOFMS, SPE-GC×GC-TOFMS, and SBSE-GC×GC-TOFMS.

Class	Number of Compounds			
	SPME	SPE	SBSE	Total
Esters	125	125	121	179
Aldehydes & Ketones	71	66	66	111
Terpenes	62	30	45	82
Alcohols	57	47	50	81
Sulfides	29	20	27	37
Furans	20	18	21	29
Nitrogenous compounds	6	23	18	29
Acids	19	18	20	23
Phenols	13	14	15	18
Lactones	7	12	12	17
Total	409	373	395	606

**Table 2:** A total of 82 terpene compounds in Chinese herbaceous aroma-type Baijiu.

NO.	Compounds	RT1 ^a^	RT2 ^b^	Similarity	LRIcal ^c^	LRIlit ^d^	Identification ^e^
1	δ-3-Carene	784	2.98	842	1139	1166	RI, MS, Tent
2	α-Limonene	892	2.8	913	1193	1200	RI, MS, STD
3	1,8-Cineole	920	2.97	862	1207	1211	RI, MS, Tent
4	Terpinolene	1124	2.69	886	1306	1280	RI, MS, STD
5	α-Thujone	1340	2.44	790	1414	1431	RI, MS, Tent
6	*trans*-Linalool oxide	1460	1.99	925	1476	1483	RI, MS, Tent
7	*cis*-Linalool oxide	1464	2.02	921	1478	1454	RI, MS, Tent
8	α-Longipinene	1472	3.59	828	1483	1482	RI, MS, Tent
9	α-Copaene	1504	3.63	831	1500	1497	RI, MS, Tent
10	Daucene	1508	3.63	879	1502	1495	RI, MS, STD
11	Longicyclene	1528	3.67	889	1513	1497	RI, MS, Tent
12	Theaspirane B	1532	3.17	717	1515	1522	RI, MS, Tent
13	Camphor	1532	3.87	758	1515	1540	RI, MS, STD
14	(−)-Camphor	1564	2.41	949	1532	1532	RI, MS, Tent
15	Vitispirane	1576	2.95	853	1539	1527	RI, MS, Tent
16	α-Gurjunene	1580	3.77	903	1541	1529	RI, MS, Tent
17	Linalool	1588	1.81	947	1545	1552	RI, MS, STD
18	Theaspirane	1600	3.11	838	1552	1523	RI, MS, Tent
19	α-Cedrene	1628	3.74	877	1568	1571	RI, MS, STD
20	Carvomenthone	1628	2.46	753	1567	1552	RI, MS, Tent
21	β-Funebrene	1636	3.7	860	1572	1588	RI, MS, Tent
22	Junipene	1656	3.61	926	1583	1583	RI, MS, Tent
23	d-Fenchyl alcohol	1664	1.87	942	1586	1588	RI, MS, Tent
24	α-*trans*-Bergamotene	1672	3.35	903	1591	1583	RI, MS, Tent
25	α-Guaiene	1684	3.48	860	1598	1598	RI, MS, Tent
26	β-Elemene	1684	3.04	908	1598	1586	RI, MS, Tent
27	Calarene	1692	3.62	916	1602	1604	RI, MS, STD
28	*trans*-Caryophyllene	1700	3.43	949	1607	1581	RI, MS, STD
29	Terpinen-4-ol	1704	2.09	940	1608	1628	RI, MS, STD
30	Isophorone	1708	2.07	920	1610	1600	RI, MS, STD
31	*trans*-Edulan	1720	2.8	748	1617	1620	RI, MS, Tent
32	β-Terpineol	1748	1.91	862	1632	1616	RI, MS, Tent
33	β-Cyclocitral	1748	2.4	824	1632	1613	RI, MS, STD
34	α-Patchoulene	1776	3.86	819	1648	1640	RI, MS, Tent
35	Alloaromadendrene	1788	3.88	884	1655	1644	RI, MS, Tent
36	β-Barbatene	1800	3.84	746	1662	1667	RI, MS, Tent
37	γ-Gurjunene	1804	3.93	919	1664	1674	RI, MS, Tent
38	Isoborneol	1820	2.03	803	1671	1672	RI, MS, Tent
39	α-Humulene	1832	3.78	919	1679	1680	RI, MS, Tent
40	l-Borneol	1852	2.05	730	1689	1675	RI, MS, Tent
41	α-Terpineol	1872	2.02	958	1700	1700	RI, MS, STD
42	γ-Amorphene	1864	3.68	895	1696	1724	RI, MS, Tent
43	Ledene	1880	3.68	902	1705	1701	RI, MS, Tent
44	*trans*-Borneol	1880	1.95	924	1704	1679	RI, MS, Tent
45	β-Chamigrene	1900	3.66	864	1716	1702	RI, MS, Tent
46	Valencene	1928	3.44	899	1731	1726	RI, MS, Tent
47	α-bisabolene	1936	3.18	878	1735	1720	RI, MS, STD
48	Germacrene A	1956	3.37	839	1747	1743	RI, MS, Tent
49	α-Chamigrene	1960	3.46	851	1749	1753	RI, MS, Tent
50	δ-Cadinene	1988	3.25	932	1764	1753	RI, MS, STD
51	β-Citronellol	1992	1.77	889	1765	1771	RI, MS, STD
52	7 epi-a-Selinene	2008	3.26	873	1775	1772	RI, MS, Tent
53	α-Curcumene	2016	2.79	881	1779	1788	RI, MS, Tent
54	Nerol	2072	1.7	845	1811	1821	RI, MS, Tent
55	Isogeraniol	2096	1.69	832	1827	1818	RI, MS, Tent
56	β-Damascenone	2104	2.26	910	1832	1827	RI, MS, STD
57	Dihydro-β-ionone	2124	2.36	835	1845	1854	RI, MS, Tent
58	l-calamenene	2124	2.81	946	1846	1838	RI, MS, STD
59	Geraniol	2132	1.7	872	1850	1851	RI, MS, STD
60	*trans*-Geranylacetone	2148	2.19	877	1861	1862	RI, MS, STD
61	Geosmin	2148	2.32	902	1861	1858	RI, MS, STD
62	α-Ionone	2156	2.2	846	1866	1866	RI, MS, STD
63	α-Dehydro-himachalene	2184	2.61	836	1885	1882	RI, MS, Tent
64	α-Calacorene	2248	2.53	898	1930	1904	RI, MS, Tent
65	Palustrol	2264	2.46	899	1941	1938	RI, MS, Tent
66	*trans*-β-Ionone	2280	2.15	854	1952	1953	RI, MS, STD
67	*cis*-Jasmone	2292	1.99	859	1961	1955	RI, MS, STD
68	β-Caryophyllene oxide	2296	2.17	792	1964	1990	RI, MS, Tent
69	d-Nerolidol	2388	1.84	921	2036	2010	RI, MS, Tent
70	*E*-Nerolidol	2392	1.82	926	2040	2054	RI, MS, Tent
71	Epicubenol	2436	2.07	765	2077	2078	RI, MS, Tent
72	α-Corocalene	2436	2.15	863	2077	2083	RI, MS, Tent
73	Cubenol	2436	2.07	787	2077	2071	RI, MS, Tent
74	6-Isocedrol	2496	1.95	894	2135	2162	RI, MS, Tent
75	α-Cedrol	2496	1.95	877	2135	2127	RI, MS, Tent
76	β-Bisabolol	2520	1.82	728	2160	2151	RI, MS, Tent
77	Torreyol	2556	1.92	815	2197	2197	RI, MS, Tent
78	α-Cadinol	2556	1.92	810	2197	2217	RI, MS, STD
79	α-Eudesmol	2592	1.98	719	2237	2223	RI, MS, Tent
80	β-Eudesmol	2600	2	821	2246	2246	RI, MS, Tent
81	Farnesol	2700	1.95	846	2353	2351	RI, MS, Tent
82	9H-Fluorene	2732	2.16	907	2386	2374	RI, MS, Tent

^a^ RT1: retention time on the primary column. ^b^ RT2: retention time on the secondary column. ^c^ LRIcal: calculated linear retention indices. ^d^ LRIlit: literature linear retention indices obtained from the NIST library (https://webbook.nist.gov/chemistry/). ^e^ Identification: tentative identification (Tent.) based on retention indices (RI) and mass spectra (MS), positive identification based on retention times of authentic standards (STD).

**Table 3:** A total of 37 sulfides in Chinese herbaceous aroma-type Baijiu.

No	Compounds	RT1 ^a^	RT2 ^b^	Similarity	LRIcal ^c^	LRIlit ^d^	Identification ^e^
1	Methanethiol	292	1.34	985	669	643	RI, MS, STD
2	Dimethyl sulfide	316	1.43	895	750	774	RI, MS, STD
3	Methyl thiolacetate	628	1.69	814	1054	1052	RI, MS, Tent
4	Dimethyl disulfide	668	1.81	960	1077	1078	RI, MS, STD
5	*S*-Methyl propanethioate	752	1.95	749	1122	1131	RI, MS, STD
6	Methyl ethyl disulfide	804	2.05	736	1149	1141	RI, MS, Tent
7	*S*-Methyl ester butanethioic acid	908	2.13	835	1201	1198	RI, MS, STD
8	Thiazole	1032	1.59	907	1261	1259	RI, MS, STD
9	Dimethyl trisulphide	1312	2.16	966	1399	1400	RI, MS, STD
10	*S*-Methyl hexanethioate	1340	2.37	895	1414	1412	RI, MS, Tent
11	Methyl pentyl disulfide	1400	2.48	764	1445	1445	RI, MS, Tent
12	4,5-Dimethyl-2-isopropyl-thiazole	1424	2.47	747	1457	1436	RI, MS, Tent
13	Ethyl 2-(methylthio)acetate	1428	1.88	902	1459	1484	RI, MS, STD
14	Methional	1448	1.72	826	1470	1480	RI, MS, STD
15	2-Pentyl-thiophene	1448	2.51	893	1470	1452	RI, MS, Tent
16	Furfuryl methyl sulfide	1504	1.87	913	1499	1492	RI, MS, Tent
17	4,5-Dimethyl-2-isobutylthiazole	1568	2.57	709	1534	1514	RI, MS, Tent
18	2-(Methylthio)ethanol	1576	1.5	725	1538	1520	RI, MS, Tent
19	Methyl propyl trisulfide	1588	2.47	752	1545	1529	RI, MS, Tent
20	Ethyl 3-(methylthio)propionate	1644	2	961	1575	1580	RI, MS, STD
21	2,5-Dimethyl-1,3,4-trithiolane	1724	2.32	865	1619	1618	RI, MS, Tent
22	3-(Methylthio)propyl acetate	1760	1.99	752	1639	1627	RI, MS, Tent
23	2,4,5-Trithiahexane	1828	2.26	895	1676	1662	RI, MS, Tent
24	Methyl benzyl sulfide	1836	2.36	932	1680	1665	RI, MS, STD
25	3-Thiophenecarboxaldehyde	1868	1.77	711	1697	1687	RI, MS, Tent
26	2-Thiophenecarboxaldehyde	1896	1.73	920	1713	1722	RI, MS, STD
27	Methionol	1916	1.56	914	1724	1721	RI, MS, STD
28	5-Methyl-2-formylthiophene	1932	1.9	814	1733	1759	RI, MS, Tent
29	Dimethyl tetrasulphide	1988	2.32	727	1763	1750	RI, MS, Tent
30	1,2,4-Trithiolane	2004	2	866	1772	1760	RI, MS, Tent
31	3-Acetylthiophene	2044	1.75	752	1794	1772	RI, MS, Tent
32	2-Acetylthiophen	2044	1.74	717	1794	1785	RI, MS, STD
33	Furfuryl methyl disulfide	2088	1.94	846	1822	1813	RI, MS, Tent
34	3-Methyl-2-thiophenecarbaldehyde	2104	1.76	798	1832	1815	RI, MS, Tent
35	1-(2-Thienyl) propanone	2144	1.8	714	1858	1840	RI, MS, Tent
36	Benzothiazole	2320	1.78	835	1981	1958	RI, MS, STD
37	2-Phenylthiophene	2476	1.76	780	2114	2124	RI, MS, STD

^a^ RT1: retention time on the primary column. ^b^ RT2: retention time on the secondary column. ^c^ LRIcal: calculated linear retention indices. ^d^ LRIlit: literature linear retention indices obtained from the NIST library (https://webbook.nist.gov/chemistry/). ^e^ Identification: tentative identification (Tent.) based on retention indices (RI) and mass spectra (MS), positive identification based on retention times of authentic standards (STD).

## Supplementary Materials

The following are available online, Table S1: Volatile compounds identified in Chinese herbaceous aroma-type Baijiu by HS-SPME-GC×GC-TOFMS, SPE-GC×GC-TOFMS and SBSE-GC×GC-TOFMS, Table S2: Peak area of 606 volatile compounds identified in Chinese herbaceous aroma-type Baijiu.
