# Supplementary material for: Three Extraction Methods in Combination with GC×GC-TOFMS for the Detailed Investigation of Volatiles in Chinese Herbaceous Aroma-Type Baijiu
Source: Molecules. 2020 Sep 27;25(19):4429. doi: 10.3390/molecules25194429 (PMC7582941; doi:10.3390/molecules25194429)
Supplement: Supplementary file 1 [file molecules-25-04429-s001.pdf]

Table S1. Volatile compounds identified in Chinese herbaceous aroma-type Baijiu by HS-SPME-GC×GC-TOFMS, SPE-GC×GC-TOFMS and SBSE-GC×GC-TOFMS.

NO.	Compounds	CAS number	RT1[a]	RT2[b]	Similarity	LRIcal[c]	LRIlit[d]	Origin[e]	Identification[f]
	**Esters**								
1	Methyl acetate	79-20-9	360	1.43	847	832	844	1	RI, MS, Tent
2	Ethyl acetate	141-78-6	416	1.57	924	904	902	1,3	RI, MS, STD
3	Ethyl propanoate	105-37-3	488	1.81	949	963	977	1,2,3	RI, MS, STD
4	Ethyl 2-methyl propanoate	97-62-1	496	1.95	914	969	961	1,2,3	RI, MS, STD
5	Propyl acetate	109-60-4	508	1.77	942	979	992	1,2,3	RI, MS, STD
6	2-Butyl acetate	105-46-4	528	1.85	918	995	985	1,2,3	RI, MS, STD
7	Methyl butanoate	623-42-7	536	1.73	841	1001	997	2	RI, MS, Tent
8	Ethyl acrylate	140-88-5	544	1.67	891	1006	992	2,3	RI, MS, Tent
9	Methyl 2-methylbutyrate	868-57-5	564	1.87	875	1017	1022	2	RI, MS, Tent
10	Isobutyl acetate	110-19-0	568	1.87	947	1020	1025	1,2,3	RI, MS, STD
11	Methyl isovalerate	556-24-1	580	1.85	890	1027	1011	2	RI, MS, Tent
12	Allyl acetate	591-87-7	600	1.63	920	1038	1023	2	RI, MS, Tent
13	Ethyl butanoate	105-54-4	620	2	897	1050	1025	1,2,3	RI, MS, STD
14	Propyl propionate	106-36-5	620	1.99	960	1050	1065	2,3	RI, MS, Tent
15	Ethyl 2-methylbutanoate	7452-79-1	632	2.17	849	1057	1053	1,2,3	RI, MS, STD
16	Butyl acetate	123-86-4	664	1.93	892	1075	1082	1,2,3	RI, MS, STD
17	Ethyl 3-methylbutanoate	108-64-5	676	2.07	910	1082	1067	1,2,3	RI, MS, STD

18	Methyl pentanoate	624-24-8	684	1.93	816	1086	1082	1,2,3	RI, MS, STD
19	Isobutyl isobutyrate	97-85-8	688	2.35	891	1089	1092	1,2	RI, MS, Tent
20	Isobutyl propanoate	540-42-1	696	2	913	1093	1090	1,2,3	RI, MS, Tent
21	Propyl butyrate	105-66-8	748	2.3	932	1120	1135	1,2,3	RI, MS, STD
22	3-Methylbutyl acetate	123-92-2	756	2.11	934	1124	1131	1,2,3	RI, MS, STD
23	sec-Butyl butyrate	819-97-6	772	2.45	856	1133	1158	1,2,3	RI, MS, Tent
24	Propyl 2-methylbutanoate	37064-20-3	784	2.51	884	1139	1150	1,2	RI, MS, Tent
25	Butyl propionate	590-01-2	788	2.21	963	1141	1159	2	RI, MS, Tent
26	1-Methylpropyl 2-methylbutanoate	869-08-9	796	2.64	825	1145	1148	1	RI, MS, Tent
27	Butyl isobutyrate	97-87-0	796	2.41	936	1145	1149	2	RI, MS, Tent
28	Propyl isovalerate	557-00-6	812	2.44	886	1153	1153	1,2,3	RI, MS, Tent
29	Isobutyl isovalerate	589-59-3	832	2.52	845	1163	1190	2	RI, MS, Tent
30	Ethyl valerate	539-82-2	836	2.13	951	1165	1159	1,2,3	RI, MS, STD
31	Ethyl 2-butenoate	10544-63-5	844	1.92	929	1169	1172	2,3	RI, MS, Tent
32	Isobutyl butyrate	539-90-2	852	2.32	900	1173	1185	1,2	RI, MS, Tent
33	Isobutyl 2-methylbutanoate	2445-67-2	856	2.57	905	1175	1179	2	RI, MS, Tent
34	Allyl butanoate	2051-78-7	860	1.99	883	1177	1180	2	RI, MS, Tent
35	Ethyl 3-methylvalerate	5870-68-8	860	2.33	844	1177	1181	2	RI, MS, Tent
36	Isopentyl isobutyrate	2050-1-3	892	2.6	923	1193	1183	1	RI, MS, Tent
37	Methyl hexanoate	106-70-7	892	2.16	834	1193	1183	1,2,3	RI, MS, Tent
38	Amyl acetate	628-63-7	900	2.05	823	1197	1195	1,2,3	RI, MS, Tent

39	Ethyl 4-methylvalerate	25415-67-2	932	2.3	849	1213	1204	1,2,3	RI, MS, Tent
40	2-Pentyl butanoate	60415-61-4	932	2.63	913	1213	1216	2,3	RI, MS, Tent
41	Isoamyl propionate	105-68-0	940	2.25	897	1217	1188	3	RI, MS, Tent
42	Butyl butyrate	109-21-7	940	2.46	954	1217	1215	1,2	RI, MS, Tent
43	Propyl valerate	141-06-0	964	2.39	792	1228	1233	1,2	RI, MS, Tent
44	2-Butyl-n-valerate	23361-74-2	988	2.6	835	1240	1224	2	RI, MS, Tent
45	Pentyl isobutyrate	2445-72-9	1012	2.58	905	1251	1241	2	RI, MS, Tent
46	Butyl isovalerate	109-19-3	1020	2.5	956	1255	1252	2	RI, MS, Tent
47	Isobutyl valerate	10588-10-0	1032	2.51	911	1261	1253	2	RI, MS, Tent
48	Ethyl hexanoate	123-66-0	1032	2.53	909	1261	1240	1,2,3	RI, MS, STD
49	1-Methylhexyl acetate	5921-82-4	1044	2.34	830	1267	1266	2	RI, MS, Tent
50	Isopentyl butanoate	106-27-4	1096	2.42	893	1292	1289	1	RI, MS, Tent
51	Hexyl acetate	142-92-7	1104	2.14	913	1296	1277	1,2,3	RI, MS, STD
52	Isopentyl 2-methylbutanoate	27625-35-0	1112	2.6	920	1300	1274	1,2,3	RI, MS, Tent
53	Methyl heptanoate	106-73-0	1124	2.22	918	1306	1327	1,3	RI, MS, Tent
54	Isopentyl isovalerate	659-70-1	1136	2.57	940	1312	1312	1,2	RI, MS, Tent
55	Ethyl (E)-3-hexenoate	26553-46-8	1148	2.16	946	1317	1303	1,2,3	RI, MS, Tent
56	Ethyl lactate	97-64-3	1180	1.51	976	1333	1309	3	RI, MS, Tent
57	Propyl hexanoate	626-77-7	1192	2.56	854	1340	1313	1,2,3	RI, MS, STD
58	(Z)-3-hexen-1-yl acetate	3681-71-8	1196	2.05	823	1341	1346	3	RI, MS, Tent
59	Ethyl 2-hydroxypropanoate	687-47-8	1200	1.51	969	1343	1356	2,3	RI, MS, Tent

60	Amyl 2-methylbutyrate	68039-26-9	1208	2.65	857	1348	1327	1,2,3	RI, MS, Tent
61	Ethyl β-ethoxypropionate	763-69-9	1208	2.03	919	1347	1320	1,2,3	RI, MS, Tent
62	Ethyl 2-hexenoate	1552-67-6	1212	2.29	892	1349	1328	2	RI, MS, STD
63	Ethyl heptanoate	106-30-9	1224	2.46	813	1355	1341	1,2,3	RI, MS, STD
64	Hexyl propanoate	2445-76-3	1228	2.44	921	1357	1353	1,2,3	RI, MS, Tent
65	Hexyl isobutyrate	2349-7-7	1232	2.63	945	1359	1342	1,2,3	RI, MS, Tent
66	Amyl isovalerate	25415-62-7	1240	2.59	922	1363	1350	1,2,3	RI, MS, Tent
67	Isobutyl hexanoate	105-79-3	1248	2.64	906	1367	1347	1,2,3	RI, MS, Tent
68	Isoamyl valerate	2050-9-1	1260	2.62	910	1373	1346	1,2,3	RI, MS, Tent
69	2-Propenyl hexanoate	123-68-2	1276	2.24	875	1381	1370	1,2	RI, MS, Tent
70	Heptyl acetate	112-06-1	1280	2.31	929	1383	1385	1,2,3	RI, MS, STD
71	Ethyl Z-4-heptenoate	54340-70-4	1288	2.29	892	1387	1377	1,3	RI, MS, Tent
72	Methyl octanoate	111-11-5	1308	2.36	916	1397	1399	1,2,3	RI, MS, Tent
73	2-Heptyl butanoate	39026-94-3	1312	2.77	856	1399	1401	2,3	RI, MS, STD
74	Cyclopentyl butyrate	6290-13-7	1324	2.54	826	1405	1387	1	RI, MS, Tent
75	Ethyl 2-hydroxybutyrate	52089-54-0	1336	1.58	858	1411	1400	1,2	RI, MS, Tent
76	Ethyl 3-hydroxy-3-methylbutyrate	18267-36-2	1340	1.7	870	1413	1400	2	RI, MS, Tent
77	Butyl hexanoate	626-82-4	1352	2.67	928	1420	1406	1,2,3	RI, MS, STD
78	Propyl heptanoate	7778-87-2	1352	2.64	947	1420	1425	2,3	RI, MS, STD
79	Hexyl butanoate	2639-63-6	1368	2.6	826	1428	1430	1,2,3	RI, MS, STD
80	Isopropyl octanoate	5458-59-3	1376	2.72	852	1433	1419	2,3	RI, MS, Tent

81	Propyl lactate	616-09-1	1376	1.58	940	1432	1424	1,2,3	RI, MS, Tent
82	Ethyl 2-hydroxy-3-methylbutyrate	2441-6-7	1380	1.68	944	1434	1427	1,2,3	RI, MS, Tent
83	Hexyl 2-methylbutyrate	10032-15-2	1384	2.77	896	1437	1431	1,2,3	RI, MS, Tent
84	Ethyl cyclohexanoate	3289-28-9	1384	2.51	933	1437	1429	1,2,3	RI, MS, Tent
85	Ethyl octoate	106-32-1	1392	2.51	919	1441	1435	1,2,3	RI, MS, STD
86	Heptyl propionate	2216-81-1	1412	2.52	818	1451	1455	1,3	RI, MS, Tent
87	Hexyl isopentanoate	10032-13-0	1416	2.7	936	1453	1425	1,2,3	RI, MS, Tent
88	Isoamyl hexanoate	2198-61-0	1428	2.77	893	1460	1453	2,3	RI, MS, STD
89	Isobutyl heptanoate	7779-80-8	1428	2.71	911	1460	1448	1	RI, MS, STD
90	Isobutyl lactate	585-24-0	1440	1.62	902	1465	1455	1,2,3	RI, MS, Tent
91	2-Methylbutyl hexanoate	2601-13-0	1444	2.9	843	1468	1458	1,2	RI, MS, Tent
92	2-Ethylhexyl 2-propenoate	103-11-7	1484	2.51	893	1489	1494	1	RI, MS, Tent
93	Ethyl 7-octenoate	35194-38-8	1488	2.35	919	1491	1478	1,2,3	RI, MS, Tent
94	Ethyl diethoxyacetate	6065-82-3	1496	2.05	929	1495	1487	1,2,3	RI, MS, Tent
95	Methyl nonanoate	1731-84-6	1500	2.45	810	1497	1481	1	RI, MS, Tent
96	Pentyl hexanoate	540-07-8	1536	2.82	859	1517	1501	1,2,3	RI, MS, STD
97	Ethyl 3-hydroxybutyrate	5405-41-4	1544	1.56	912	1520	1522	1,2	RI, MS, Tent
98	Heptyl butanoate	5870-93-9	1544	2.69	885	1521	1522	1	RI, MS, Tent
99	Butyl 2-hydroxypropanoate	138-22-7	1552	1.64	894	1525	1520	1,2,3	RI, MS, STD
100	Propyl octanoate	624-13-5	1552	2.69	919	1525	1514	1,2,3	RI, MS, STD
101	Heptyl 2-methylbutanoate	50862-12-9	1560	2.88	836	1530	1516	1	RI, MS, Tent

102	Ethyl nonanoate	123-29-5	1580	2.62	943	1541	1526	1,2,3	RI, MS, STD
103	Octyl propanoate	142-60-9	1588	2.63	915	1545	1539	1	RI, MS, Tent
104	Ethyl 2-hydroxy-4-methylpentanoate	10348-47-7	1592	1.74	913	1547	1547	1,3	RI, MS, Tent
105	Ethyl E-2-octenoate	7367-82-0	1612	2.42	901	1558	1540	3	RI, MS, Tent
106	Isopentyl heptanoate	109-25-1	1616	2.82	942	1561	1552	3	RI, MS, Tent
107	Nonyl acetate	143-13-5	1652	2.5	873	1580	1593	1	RI, MS, Tent
108	Ethyl 3-acetoxybutanoate	27846-49-7	1656	1.88	849	1582	1570	3	RI, MS, Tent
109	Diethyl propanedioate	105-53-3	1660	1.82	829	1584	1595	1,2,3	RI, MS, STD
110	2-Heptyl hexanoate	6624-58-4	1668	2.94	904	1589	1599	1,2,3	RI, MS, Tent
111	2-Camphanol acetate	76-49-3	1668	2.67	932	1589	1590	3	RI, MS, Tent
112	3-methyl-2-butenyl hexanoate	76649-22-4	1672	2.54	852	1591	1572	1	RI, MS, Tent
113	Methyl decanoate	110-42-9	1684	2.52	922	1597	1585	1,3	RI, MS, Tent
114	Hexyl hexanoate	6378-65-0	1712	2.85	846	1613	1599	1,2,3	RI, MS, STD
115	Ethyl levulate	539-88-8	1724	1.8	890	1619	1610	1,2,3	RI, MS, Tent
116	Octyl butanoate	110-39-4	1728	2.72	808	1622	1624	1,2	RI, MS, Tent
117	Butyl octanoate	589-75-3	1728	2.76	829	1622	1619	1,2	RI, MS, Tent
118	Octyl 2-methylbutanoate	29811-50-5	1740	2.97	820	1628	1623	1	RI, MS, Tent
119	Methyl benzoate	93-58-3	1756	1.96	905	1636	1637	1,2,3	RI, MS, Tent
120	Ethyl decanoate	110-38-3	1760	2.87	868	1639	1648	1,2,3	RI, MS, STD
121	Ethyl methyl butanedioate	627-73-6	1760	1.88	909	1639	1631	2,3	RI, MS, Tent
122	Diethyl fumarate	623-91-6	1788	2.03	915	1654	1647	1,2,3	RI, MS, Tent

123	Isoamyl octanoate	2035-99-6	1796	3.2	941	1659	1658	1,2,3	RI, MS, Tent
124	Ethyl trans-4-decenoate	76649-16-6	1808	2.84	927	1665	1680	1,3	RI, MS, STD
125	Isopropyl benzoate	939-48-0	1808	2.3	876	1665	1676	1,3	RI, MS, Tent
126	Diethyl succinate	123-25-1	1832	2.1	970	1678	1694	1,2,3	RI, MS, Tent
127	Ethyl benzoate	93-89-0	1832	2.22	970	1678	1652	1,2,3	RI, MS, STD
128	Ethyl 3-hydroxyhexanoate	2305-25-1	1836	1.85	850	1680	1652	2	RI, MS, Tent
129	Ethyl cis-4-decenoate	7367-84-2	1844	2.89	888	1685	1699	1	RI, MS, Tent
130	Ethyl 9-decenoate	67233-91-4	1856	2.81	793	1692	1703	1	RI, MS, STD
131	α-Terpineol acetate	80-26-2	1876	2.66	871	1702	1700	1	RI, MS, STD
132	α-Phenylethyl acetate	93-92-5	1892	2.07	861	1711	1693	3	RI, MS, Tent
133	Propyl decanoate	30673-60-0	1928	2.94	908	1731	1720	2,3	RI, MS, Tent
134	Benzyl acetate	140-11-4	1948	1.95	907	1741	1762	1,2,3	RI, MS, Tent
135	Ethyl undecanoate	627-90-7	1948	2.84	941	1742	1744	1,2,3	RI, MS, STD
136	Butyl butyrolactate	7492-70-8	1956	2.35	830	1746	1733	1	RI, MS, Tent
137	Ethyl trans-2-decenoate	37486-72-9	1992	2.55	881	1766	1750	1	RI, MS, Tent
138	Propyl benzoate	2315-68-6	2000	2.15	937	1770	1777	1	RI, MS, Tent
139	Methyl phenylacetate	101-41-7	2004	1.93	832	1772	1758	1,2,3	RI, MS, Tent
140	Ethyl glutarate	818-38-2	2032	1.96	855	1787	1768	2,3	RI, MS, Tent
141	Benzyl isobutanoate	103-28-6	2036	2.12	865	1790	1797	1	RI, MS, Tent
142	Methyl salicylate	119-36-8	2044	1.88	814	1794	1781	3	RI, MS, STD
143	Ethyl phenylacetate	101-97-3	2048	2	894	1796	1785	1,3	RI, MS, STD

144	Isobutyl benzoate	120-50-3	2056	2.17	926	1801	1806	1,2,3	RI, MS, Tent
145	Benzyl propionate	122-63-4	2060	1.99	932	1803	1784	1,3	RI, MS, Tent
146	Hexyl octanoate	1117-55-1	2080	2.74	877	1817	1804	1,2,3	RI, MS, STD
147	β-Phenethyl acetate	103-45-7	2096	1.94	972	1827	1835	1,2,3	RI, MS, Tent
148	Ethyl dodecanoate	106-33-2	2128	2.61	940	1848	1860	1,2,3	RI, MS, STD
149	Methyl benzenepropanoate	103-25-3	2148	1.91	875	1861	1857	1,3	RI, MS, Tent
150	n-Butyl benzoate	136-60-7	2172	2.08	824	1877	1879	1	RI, MS, Tent
151	Benzyl butanoate	103-37-7	2184	1.99	945	1884	1856	3	RI, MS, Tent
152	β-Phenylethyl isobutyrate	103-48-0	2196	2.07	890	1892	1900	3	RI, MS, Tent
153	Ethyl 3-phenylpropionate	2021-28-5	2204	2.04	873	1898	1900	1,2,3	RI, MS, STD
154	Propyl dodecanoate	3681-78-5	2244	2.56	832	1927	1931	1,3	RI, MS, Tent
155	Isoamyl benzoate	94-46-2	2244	2.09	931	1926	1928	1,3	RI, MS, Tent
156	Benzenepropyl acetate	122-72-5	2284	1.9	868	1955	1971	1,3	RI, MS, STD
157	Butyl phenylacetate	122-43-0	2304	2.01	876	1970	1970	1,2,3	RI, MS, STD
158	2-Phenylethyl butanoate	103-52-6	2312	2.01	927	1976	1968	1,3	RI, MS, Tent
159	Phenethyl 2-methylbutyrate	24817-51-4	2320	2.08	862	1981	1968	1	RI, MS, Tent
160	Phenethyl isovalerate	140-26-1	2344	2.04	905	1999	1986	1,2,3	RI, MS, STD
161	Ethyl tetradecanoate	124-06-1	2400	2.27	933	2047	2053	2,3	RI, MS, STD
162	Diethyl dl-malate	626-11-9	2404	1.5	917	2050	2062	2	RI, MS, Tent
163	Triacetyl glycerol	102-76-1	2440	1.53	973	2080	2077	2	RI, MS, Tent
164	Diethyl octanedioate	2050-23-9	2472	1.78	842	2110	2114	2,3	RI, MS, Tent

165	Ethyl cinnamate	103-36-6	2512	1.68	947	2151	2156	1,2,3	RI, MS, STD
166	Ethyl pentadecanoate	41114-00-5	2512	2.09	916	2152	2140	1,2,3	RI, MS, STD
167	Hexyl phenylacetate	5421-17-0	2536	1.88	910	2176	2148	1,2	RI, MS, Tent
168	Methyl hexadecanoate	112-39-0	2572	2.21	918	2215	2245	3	RI, MS, Tent
169	Ethyl hexadecanoate	628-97-7	2604	2.41	949	2251	2240	1,2,3	RI, MS, STD
170	Ethyl hexadec-9-enoate	54546-22-4	2632	2.44	894	2282	2267	2,3	RI, MS, Tent
171	Hexyl dihydrocinnamate	220766-75-6	2644	2.15	868	2295	2296	1	RI, MS, Tent
172	Ethyl heptadecanoate	14010-23-2	2668	2.66	832	2321	2340	2,3	RI, MS, Tent
173	Propyl hexadecanoate	2239-78-3	2684	2.75	833	2337	2335	3	RI, MS, Tent
174	Ethyl hydrogen succinate	1070-34-4	2732	1.36	900	2386	2395	1,2	RI, MS, Tent
175	Butyl hexadecanoate	111-06-8	2776	3.25	882	2430	2419	3	RI, MS, Tent
176	Ethyl octadecanoate	111-61-5	2800	3.3	939	2453	2483	3	RI, MS, Tent
177	Ethyl cis-9-octadecenoate	111-62-6	2824	3.37	882	2476	2476	2,3	RI, MS, Tent
178	Ethyl linoleate	544-35-4	2880	3.5	936	2526	2521	2,3	RI, MS, Tent
179	Ethyl vanillate	617-05-0	3056	2	873	2656	2668	2,3	RI, MS, Tent
	**Alcohols**								
180	2-Propanol	67-63-0	440	1.4	963	924	935	1	RI, MS, STD
181	2-Butanol	78-92-2	584	1.48	934	1029	1048	1,2,3	RI, MS, STD
182	1-Propanol	71-23-8	612	1.45	934	1045	1046	1,3	RI, MS, STD
183	2-Methyl-3-buten-2-ol	115-18-4	620	1.42	826	1049	1036	2	RI, MS, Tent
184	2-Methyl-1-propanol	78-83-1	716	1.48	961	1104	1108	1,2,3	RI, MS, STD

185	3-Pentanol	584-02-1	740	1.52	887	1116	1097	2	RI, MS, Tent
186	2-Pentanol	6032-29-7	764	1.52	957	1128	1130	2,3	RI, MS, STD
187	1-Butanol	71-36-3	800	1.47	954	1146	1120	1,2,3	RI, MS, STD
188	2-Methyl-3-pentanol	565-67-3	824	1.61	813	1158	1167	1	RI, MS, Tent
189	3-Penten-2-ol	1569-50-2	852	1.46	916	1172	1163	2	RI, MS, Tent
190	3-Hexanol	623-37-0	904	1.59	834	1199	1206	2	RI, MS, STD
191	2-Methylbutanol	137-32-6	920	1.52	957	1207	1197	1,2	RI, MS, STD
192	3-Methyl-1-butanol	123-51-3	944	1.49	951	1218	1208	1,2,3	RI, MS, STD
193	2-Hexanol	626-93-7	956	1.58	950	1224	1226	1,2,3	RI, MS, STD
194	3-Methyl-3-buten-1-ol	763-32-6	1020	1.56	831	1255	1274	1,2	RI, MS, STD
195	1-Pentanol	71-41-0	1024	1.61	975	1257	1256	1,2,3	RI, MS, STD
196	4-Heptanol	589-55-9	1104	1.67	825	1295	1290	1,2,3	RI, MS, Tent
197	2-(Z)-Pentenol	1576-95-0	1120	1.43	823	1303	1322	2	RI, MS, STD
198	(S)-2-Heptanol	6033-23-4	1120	1.66	952	1303	1307	3	RI, MS, Tent
199	2-Ethyl-1-butanol	97-95-0	1124	1.55	930	1305	1318	2,3	RI, MS, Tent
200	2-Methyl-1-pentanol	105-30-6	1136	1.52	846	1311	1337	3	RI, MS, Tent
201	Cyclopentanol	96-41-3	1136	1.52	906	1311	1298	1,2	RI, MS, STD
202	Prenol	556-82-1	1156	1.46	927	1321	1325	2,3	RI, MS, Tent
203	4-Methyl-1-pentanol	626-89-1	1156	1.52	931	1321	1338	1,3	RI, MS, STD
204	2-Heptanol	543-49-7	1164	1.65	959	1325	1327	1,2,3	RI, MS, STD
205	3-Methyl-1-pentanol	589-35-5	1176	1.53	907	1331	1344	1,2,3	RI, MS, STD

206	3-Methyl-cyclopentanol	18729-48-1	1192	1.57	874	1339	1342	2	RI, MS, Tent
207	1-Hexanol	111-27-3	1236	1.7	879	1361	1356	1,2,3	RI, MS, STD
208	3-Hexen-1-ol	544-12-7	1252	1.52	873	1369	1384	1	RI, MS, Tent
209	5-Methyl-2-heptanol	54630-50-1	1264	1.68	898	1375	1394	3	RI, MS, STD
210	3-Ethoxy-1-propanol	111-35-3	1276	1.53	880	1381	1376	1,2	RI, MS, Tent
211	4-Octanol	589-62-8	1280	1.75	926	1383	1376	1,3	RI, MS, Tent
212	4-Methyl-3-penten-1-ol	763-89-3	1288	1.52	874	1387	1390	2,3	RI, MS, Tent
213	3-Octanol	589-98-0	1300	1.76	963	1393	1394	1,2,3	RI, MS, STD
214	Cyclohexanol	108-93-0	1320	1.62	832	1403	1403	2	RI, MS, Tent
215	2-Butoxy-ethanol	111-76-2	1328	1.58	862	1407	1402	1,2,3	RI, MS, Tent
216	cis-2-Hexenol	928-94-9	1328	1.61	807	1407	1403	3	RI, MS, STD
217	3-Methyl-1-hexanol	13231-81-7	1336	1.6	889	1411	1413	1,3	RI, MS, Tent
218	5-Hexen-1-ol	821-41-0	1336	1.51	857	1411	1394	1	RI, MS, STD
219	4-Methyl-1-hexanol	818-49-5	1376	1.61	909	1432	1434	1,2,3	RI, MS, STD
220	1-Octen-3-ol	3391-86-4	1412	1.66	974	1451	1462	1,2,3	RI, MS, STD
221	1-HeptanoI	111-70-6	1420	1.65	959	1455	1440	1,2,3	RI, MS, STD
222	6-Methyl-5-hepten-2-ol	1569-60-4	1436	1.68	837	1463	1464	1,2,3	RI, MS, STD
223	4-Nonanol	5932-79-6	1464	1.85	916	1478	1467	1,3	RI, MS, STD
224	2-Ethyl-1-hexanol	104-76-7	1484	1.7	959	1488	1484	1,2,3	RI, MS, STD
225	4-Hepten-1-ol	20851-55-2	1512	1.58	817	1503	1502	1,2	RI, MS, STD
226	(E)-2-Hepten-1-ol	33467-76-4	1524	1.57	822	1510	1504	1,2,3	RI, MS, STD

227	2-Nonanol	628-99-9	1536	1.81	931	1516	1489	1,2,3	RI, MS, STD
228	1-Octanol	111-87-5	1608	1.72	947	1556	1559	1,2,3	RI, MS, STD
229	p-Menthan-8-ol	498-81-7	1624	1.99	863	1564	1569	3	RI, MS, Tent
230	5-Decanol	5205-34-5	1640	1.93	828	1573	1574	1	RI, MS, Tent
231	[S,S]-2,3-Butanediol	19132-06-0	1648	1.34	859	1577	1581	1	RI, MS, Tent
232	(Z)-3-Octen-1-ol	20125-84-2	1660	1.66	836	1584	1563	3	RI, MS, STD
233	(Z)-5-Octen-1-ol	64275-73-6	1696	1.66	801	1604	1608	1	RI, MS, Tent
234	(E)-2-Octen-1-ol	18409-17-1	1712	1.64	903	1612	1590	1,2	RI, MS, Tent
235	2-Octen-1-ol	22104-78-5	1712	1.64	891	1612	1637	3	RI, MS, STD
236	2,6-Dimethyl-5-hepten-1-ol	4234-93-9	1780	1.76	835	1649	1654	1	RI, MS, Tent
237	1-Nonanol	143-08-8	1788	1.84	939	1654	1662	1,2,3	RI, MS, STD
238	6-Undecanol	23708-56-7	1812	2.16	846	1667	1640	1	RI, MS, Tent
239	Undecan-4-ol	4272-6-4	1820	2.16	908	1672	1672	1	RI, MS, Tent
240	(Z)-3-Nonen-1-ol	10340-23-5	1844	1.84	877	1684	1682	2,3	RI, MS, Tent
241	(E)-2-Nonen-1-ol	31502-14-4	1896	1.75	908	1713	1715	1,3	RI, MS, Tent
242	2-Undecanol	1653-30-1	1900	2.02	963	1715	1738	1,3	RI, MS, Tent
243	cis-6-Nonen-1-ol	35854-86-5	1900	1.77	872	1715	1714	1,3	RI, MS, Tent
244	1-Decanol	112-30-1	1984	1.83	933	1761	1771	1,2,3	RI, MS, STD
245	1-Methyl-1-phenylethanol	617-94-7	1988	1.66	877	1763	1776	2,3	RI, MS, Tent
246	(Z)-4-Decen-1-ol	57074-37-0	2036	1.77	950	1789	1784	1	RI, MS, Tent
247	(Z)-3-Decen-1-ol	10340-22-4	2052	1.74	852	1798	1783	3	RI, MS, Tent

248	α-Phenylethyl alcohol	98-85-1	2084	1.55	949	1819	1820	2,3	RI, MS, Tent
249	2-Decen-1-ol	22104-80-9	2088	1.71	906	1821	1812	1	RI, MS, Tent
250	p-Cymen-8-ol	1197-01-9	2144	1.67	816	1858	1844	1,2,3	RI, MS, Tent
251	1-Undecanol	112-42-5	2148	1.8	868	1861	1850	1	RI, MS, Tent
252	Benzenemethanol	100-51-6	2188	1.47	942	1887	1895	1,2,3	RI, MS, STD
253	α-Ethylbenzyl alcohol	93-54-9	2188	1.59	803	1887	1908	3	RI, MS, Tent
254	trans-2-Undecen-1-ol	75039-84-8	2232	1.72	909	1917	1899	1	RI, MS, Tent
255	Phenylethyl Alcohol	1960-12-8	2240	1.55	955	1923	1931	1,2,3	RI, MS, STD
256	6,10-Dimethyl-5,9-undecadien-2-ol	53837-34-6	2280	1.85	718	1952	1954	1	RI, MS, Tent
257	1-Dodecanol	112-53-8	2296	1.8	917	1964	1935	1	RI, MS, STD
258	Diethylene glycol	111-46-6	2320	1.35	927	1981	1989	1,2,3	RI, MS, Tent
259	Benzenepropanol	122-97-4	2412	1.53	826	2056	2061	2	RI, MS, Tent
260	1-Hexadecanol	36653-82-4	2716	2.13	907	2370	2377	1	RI, MS, Tent
	**Acids**								
261	Acetic acid	64-19-7	1416	1.28	969	1453	1465	1,2,3	RI, MS, STD
262	Formic acid	64-18-6	1548	1.23	962	1522	1510	1,2,3	RI, MS, STD
263	Propanoic acid	1979-9-4	1580	1.3	969	1540	1535	1,2,3	RI, MS, STD
264	2-Methyl-propanoic acid	79-31-2	1628	1.33	935	1566	1574	1,2,3	RI, MS, STD
265	Butanoic acid	107-92-6	1736	1.36	941	1625	1628	1,2,3	RI, MS, STD
266	2-Methyl butyric acid	116-53-0	1812	1.39	839	1667	1664	2	RI, MS, STD
267	Pentanoic acid	109-52-4	1940	1.39	953	1737	1733	1,2,3	RI, MS, STD

268	2-Methyl-pentanoic acid	97-61-0	1992	1.35	931	1765	1775	1,2,3	RI, MS, STD
269	4-Methyl-pentanoic acid	646-07-1	2056	1.34	829	1800	1800	1,2,3	RI, MS, STD
270	Hexanoic acid	142-62-1	2132	1.38	861	1850	1857	1,2,3	RI, MS, STD
271	5-Methyl-hexanoic acid	628-46-6	2216	1.36	925	1905	1914	2,3	RI, MS, STD
272	2-Ethyl-hexanoic acid	149-57-5	2272	1.38	925	1946	1950	1,2,3	RI, MS, STD
273	Heptanoic acid	111-14-8	2280	1.39	943	1952	1960	1,2,3	RI, MS, STD
274	(E)-2-Hexenoic acid	13419-69-7	2304	1.33	907	1969	1967	1	RI, MS, STD
275	Octanoic acid	124-07-2	2416	1.38	953	2060	2070	1,2,3	RI, MS, STD
276	(E,E)-2,4-Hexadienoic acid	110-44-1	2516	1.3	889	2155	2150	1	RI, MS, Tent
277	Nonanoic acid	112-05-0	2524	1.36	862	2163	2169	1,2,3	RI, MS, STD
278	Decanoic acid	334-48-5	2620	1.44	910	2267	2279	1,2,3	RI, MS, STD
279	Benzoic acid	65-85-0	2800	1.4	922	2451	2446	1,2,3	RI, MS, STD
280	Benzeneacetic acid	103-82-2	2940	1.47	919	2574	2565	1,3	RI, MS, STD
281	Tetradecanoic acid	544-63-8	3104	1.89	865	2688	2716	1,2,3	RI, MS, STD
282	Hexadecanoic acid	1957-10-3	3544	2.43	892	2929	2913	3	RI, MS, STD
283	9-Hexadecenoic acid	2091-29-4	3648	2.45	859	2986	2957	3	RI, MS, Tent
	**Aldehydes & ketones**								
284	Acetaldehyde	75-07-0	300	1.35	959	709	718	1,3	RI, MS, STD
285	Propanal	123-38-6	336	1.42	959	800	810	1	RI, MS, STD
286	2-Propanone	67-64-1	348	1.42	936	816	813	1	RI, MS, Tent
287	2-Methyl-propanal	78-84-2	348	1.49	949	817	842	1	RI, MS, Tent

288	2-Propenal	107-02-8	360	1.41	953	832	838	3	RI, MS, Tent
289	Butanal	123-72-8	392	1.54	943	875	854	1	RI, MS, STD
290	1,1-Diethoxy-ethane	105-57-7	404	1.88	898	891	910	1,3	RI, MS, STD
291	3-Methyl-butanal	590-86-3	432	1.69	936	917	916	1,3	RI, MS, STD
292	3-Buten-2-one	78-94-4	468	1.52	960	946	948	3	RI, MS, Tent
293	2,4,5-Trimethyl-1,3-dioxolane	3299-32-9	480	1.78	883	956	967	1,3	RI, MS, Tent
294	1,1-Diethoxy-propane	4744-8-5	488	2.15	900	963	950	1,3	RI, MS, STD
295	2,3-Butanedione	431-03-8	508	1.49	983	979	977	3	RI, MS, STD
296	1,1-Diethoxy-2-methyl-propane	1741-41-9	512	2.3	838	983	969	1,2,3	RI, MS, STD
297	2-Pentanone	107-87-9	516	1.72	929	985	1003	1,2	RI, MS, STD
298	Pentanal	110-62-3	516	1.74	819	985	979	1,2	RI, MS, STD
299	2-Methyl-1-buten-3-one	814-78-8	548	1.63	958	1008	1006	2,3	RI, MS, Tent
300	4-Methyl-2-pentanone	108-10-1	564	1.78	915	1017	1039	2	RI, MS, Tent
301	1,1-Diethoxy-butane	3658-95-5	616	2.43	871	1048	1031	2	RI, MS, Tent
302	2-Butenal	4170-30-3	632	1.56	918	1056	1042	2,3	RI, MS, Tent
303	1,1-Diethoxy-3-methyl-butane	3842-3-3	664	2.58	913	1075	1062	1,2,3	RI, MS, Tent
304	2-Hexanone	591-78-6	688	1.83	906	1089	1098	2	RI, MS, STD
305	2,4,6-Trimethyl-1,3,5-trioxane	123-63-7	700	1.73	896	1095	1070	2,3	RI, MS, Tent
306	Hexanal	66-25-1	700	1.9	924	1095	1114	1,2,3	RI, MS, STD
307	2-Methyl-2-butenal	1115-11-3	712	1.76	829	1102	1076	1,2	RI, MS, Tent
308	E-2-Methyl-2-butenal	497-03-0	716	1.75	826	1104	1088	3	RI, MS, Tent

309	4-Heptanone	123-19-3	764	2.12	861	1128	1118	2	RI, MS, Tent
310	(E)-3-Penten-2-one	3102-33-8	784	1.69	884	1138	1121	2	RI, MS, Tent
311	4-Methyl-3-penten-2-one	141-79-7	788	1.84	920	1140	1131	2	RI, MS, Tent
312	5-Methyl-2-hexanone	110-12-3	800	1.97	915	1147	1155	2	RI, MS, Tent
313	2-Methyl-2-pentenal	623-36-9	820	1.89	868	1157	1155	2	RI, MS, Tent
314	Acetyl valeryl	1996-4-8	832	1.86	933	1163	1153	1,2,3	RI, MS, Tent
315	Heptanal	111-71-7	884	2.09	828	1189	1178	1,2,3	RI, MS, STD
316	2-Heptanone	110-43-0	904	2	812	1199	1213	1,2,3	RI, MS, STD
317	Cyclopentanone	120-92-3	904	1.86	894	1199	1200	1,2,3	RI, MS, STD
318	3-Ethoxy-propanal	2806-85-1	908	1.71	839	1201	1185	1	RI, MS, Tent
319	2-Methyl-cyclopentanone	1120-72-5	912	1.99	854	1203	1220	2	RI, MS, Tent
320	1,1-Diethoxy-hexane	3658-93-3	1004	2.81	873	1248	1230	2,3	RI, MS, STD
321	1-(1-Ethoxyethoxy)-hexane	54484-73-0	1084	2.6	836	1286	1258	1,2	RI, MS, STD
322	3-Hydroxy-2-butanone	513-86-0	1108	1.44	950	1297	1277	1,3	RI, MS, STD
323	1,1,3-Triethoxy-dutane	5870-82-6	1112	2.55	915	1300	1310	2	RI, MS, Tent
324	Cyclohexanone	108-94-1	1116	2.04	875	1301	1311	2	RI, MS, STD
325	1,1,3-Triethoxy-propane	7789-92-6	1124	2.46	933	1306	1299	2,3	RI, MS, Tent
326	2-Octanone	111-13-7	1128	2.1	918	1307	1309	1,2,3	RI, MS, STD
327	Octanal	124-13-0	1132	2.16	886	1310	1319	1,2,3	RI, MS, STD
328	4-Nonanone	4485-09-0	1176	2.39	866	1332	1356	2	RI, MS, Tent
329	1,1-Diethoxy-heptane	688-82-4	1176	2.88	810	1332	1332	2	RI, MS, Tent

330	(E)-2-Heptenal	18829-55-5	1180	1.96	901	1333	1314	2	RI, MS, STD
331	2,2,6-Trimethyl-cyclohexanone	2408-37-9	1196	2.39	884	1341	1333	3	RI, MS, Tent
332	Ethyl orthoformate	122-51-0	1200	2.89	801	1344	1347	1	RI, MS, Tent
333	(Z)-2-Heptenal	57266-86-1	1204	1.94	901	1345	1339	1,3	RI, MS, STD
334	2-Ethyl-2-hexenal	645-62-5	1224	2.12	829	1355	1330	3	RI, MS, Tent
335	6-Methyl-5-heptene-2-one	110-93-0	1228	2.04	875	1357	1342	1,2,3	RI, MS, Tent
336	2-Nonanone	821-55-6	1240	2.21	806	1363	1367	1,2	RI, MS, STD
337	2-Cyclopenten-1-one	930-30-3	1256	1.72	948	1371	1383	2,3	RI, MS, Tent
338	2-Isopropyl-5-methyl-2-hexenal	35158-25-9	1268	2.35	877	1377	1378	1,3	RI, MS, Tent
339	2-Methyl-2-cyclopenten-1-one	1120-73-6	1276	1.87	911	1381	1395	2,3	RI, MS, Tent
340	Nonanal	124-19-6	1320	2.29	968	1403	1422	1,2,3	RI, MS, STD
341	3-Octen-2-one	1669-44-9	1352	2.05	877	1420	1408	1,3	RI, MS, Tent
342	1,1-Diethoxy-octane	54889-48-4	1376	2.93	822	1433	1431	1	RI, MS, Tent
343	2-Octenal	2363-89-5	1396	2.06	964	1443	1445	1,2	RI, MS, STD
344	(E)-2-Octenal	2548-87-0	1396	2.05	921	1443	1433	3	RI, MS, STD
345	2-Nonen-4-one	32064-72-5	1488	2.18	839	1491	1470	1	RI, MS, Tent
346	2-Decanone	693-54-9	1508	2.32	948	1501	1480	1,2	RI, MS, STD
347	Decanal	112-31-2	1516	2.37	921	1506	1506	1,2,3	RI, MS, STD
348	3-Nonen-2-one	14309-57-0	1544	2.13	862	1521	1520	1,2,3	RI, MS, Tent
349	1,1-Diethoxy-nonane	54815-13-3	1556	3.02	857	1528	1522	1,2,3	RI, MS, Tent
350	6-Undecanone	927-49-1	1568	2.56	866	1534	1527	1	RI, MS, Tent

351	Benzaldehyde	100-52-7	1596	1.75	810	1549	1568	1,2,3	RI, MS, STD
352	2,3-Dimethyl-2-cyclopentenone	1121-05-7	1608	1.98	824	1556	1535	2	RI, MS, Tent
353	3-Undecanone	2216-87-7	1636	2.5	795	1571	1586	1	RI, MS, Tent
354	3,5-(E, E)-Octadien-2-one	30086-02-3	1656	1.91	815	1582	1569	1	RI, MS, Tent
355	(E, Z)-2,6-Nonadienal	557-48-2	1684	2.03	904	1597	1591	1,3	RI, MS, STD
356	2-Undecanone	112-12-9	1696	2.4	953	1604	1592	1,2,3	RI, MS, STD
357	(3E)-6-Methyl-3,5-heptadien-2-one	16647-04-4	1700	1.91	873	1606	1582	3	RI, MS, Tent
358	Undecanal	112-44-7	1708	2.45	868	1611	1609	1	RI, MS, Tent
359	1,1-Diethoxydecane	34764-02-8	1732	3.09	823	1624	1622	1,2	RI, MS, Tent
360	2-Methyl-Benzaldehyde	529-20-4	1756	1.93	846	1636	1622	3	RI, MS, Tent
361	3-Methyl-Benzaldehyde	620-23-5	1756	1.94	867	1636	1624	1,2,3	RI, MS, STD
362	4-Methyl-Benzaldehyde	104-87-0	1764	1.92	918	1641	1654	1,2	RI, MS, Tent
363	(E)-2-Decenal	3913-81-3	1780	2.31	836	1650	1630	2	RI, MS, STD
364	(Z)-2-Decenal	2497-25-8	1784	2.31	928	1652	1630	1,3	RI, MS, Tent
365	Phenylethanal	122-78-1	1796	1.88	945	1658	1650	1,2,3	RI, MS, STD
366	Acetophenone	98-86-2	1812	1.97	953	1667	1655	1,2,3	RI, MS, STD
367	Cryptone	500-02-7	1848	2.36	815	1687	1668	3	RI, MS, Tent
368	6,10-Dimethyl-2-undecanone	1604-34-8	1852	2.84	849	1689	1663	1	RI, MS, Tent
369	Carvotanacetone	499-71-8	1860	2.52	927	1694	1697	1,2,3	RI, MS, STD
370	2-Hydroxy-benzaldehyde	1990-2-8	1864	1.82	840	1695	1699	1,2,3	RI, MS, STD
371	(E, E)-2,4-Nonadienal	5910-87-2	1896	2.05	941	1713	1686	1,2,3	RI, MS, STD

372	Dodecanal	112-54-9	1900	2.64	944	1715	1709	1	RI, MS, Tent
373	(2,2-Diethoxyethyl)-benzene	6314-97-2	1916	2.47	880	1724	1711	1,2,3	RI, MS, Tent
374	4-Ethyl-benzaldehyde	4748-78-1	1920	2.05	876	1726	1730	1,3	RI, MS, Tent
375	p-Menth-1-en-7-al	21391-98-0	1944	2.37	842	1739	1710	1	RI, MS, Tent
376	1-Phenyl-1-propanone	93-55-0	1948	2	911	1741	1734	1,2,3	RI, MS, Tent
377	p-Menth-1-en-3-one	89-81-6	1952	2.26	893	1744	1730	1,3	RI, MS, Tent
378	4-(1-Methylethyl)-benzaldehyde	122-03-2	1980	2.05	864	1759	1785	1,2	RI, MS, Tent
379	1-(4-Methylphenyl)-ethanone	122-00-9	2040	1.91	891	1792	1794	2	RI, MS, Tent
380	1-Phenyl-1-butanone	495-40-9	2072	2	945	1811	1793	1,2,3	RI, MS, Tent
381	4-Phenyl-2-butanone	2550-26-7	2172	1.85	842	1876	1851	1,2,3	RI, MS, STD
382	5-(2-Propenyl)-1,3-benzodioxole	94-59-7	2196	1.89	928	1892	1874	2,3	RI, MS, Tent
383	4-Hydroxy-2-methylacetophenone	875-59-2	2272	1.8	834	1946	1942	3	RI, MS, Tent
384	2-Phenyl-2-butenal	4411-89-6	2280	1.81	867	1952	1933	1,2,3	RI, MS, STD
385	4-Methyl-2-phenyl-2-pentenal	26643-91-4	2284	1.91	863	1955	1932	1,3	RI, MS, Tent
386	Maltol	118-71-8	2328	1.51	861	1987	1968	3	RI, MS, Tent
387	2-Pentadecanone	2345-28-0	2372	2.17	915	2023	2028	1	RI, MS, Tent
388	5-Methyl-2-phenyl-2-hexenal	21834-92-4	2396	1.85	790	2043	2052	1,3	RI, MS, Tent
389	6,10,14-Trimethyl-2-pentadecanone	502-69-2	2488	2.04	826	2127	2110	2,3	RI, MS, Tent
390	1-(4-Methoxyphenyl)-ethanone	100-06-1	2532	1.61	878	2172	2144	1,3	RI, MS, Tent
391	1-(3-Methoxyphenyl)-ethanone	586-37-8	2532	1.61	928	2172	2148	2	RI, MS, Tent
392	1-(4-Methoxyphenyl)-2-propanone	122-84-9	2540	1.63	894	2180	2170	1,3	RI, MS, Tent

393	4-Methoxy-6-(2-propenyl)-1,3-benzodioxole	607-91-0	2636	1.84	807	2286	2257	2	RI, MS, Tent
394	1-(2-Hydroxy-4-methoxyphenyl)-ethanone	552-41-0	2656	1.77	862	2307	2300	1,2,3	RI, MS, Tent
	**Furans**								
395	Furan	110-00-9	336	1.39	863	800	798	1	RI, MS, STD
396	2-Methyl-furan	534-22-5	388	1.51	939	870	875	1,3	RI, MS, STD
397	2-Ethyl-furan	3208-16-0	480	1.7	748	956	960	1	RI, MS, Tent
398	Dihydro-2-methyl-3(2H)-furanone	3188-00-9	1060	1.71	830	1274	1270	2	RI, MS, STD
399	3-Furaldehyde	498-60-2	1388	1.51	936	1438	1438	2,3	RI, MS, STD
400	2-Furaldehyde diethyl acetal	13529-27-6	1452	2.02	909	1472	1456	1	RI, MS, STD
401	2-Furancarboxaldehyde	1998-1-1	1464	1.57	967	1478	1486	1,2,3	RI, MS, STD
402	Benzofuran	271-89-6	1536	1.85	872	1516	1496	2,3	RI, MS, Tent
403	1-(2-Furanyl)-ethanone	1192-62-7	1544	1.63	941	1521	1498	1,2,3	RI, MS, STD
404	Tetrahydro-2-furanmethanol	97-99-4	1548	1.9	733	1523	1501	1	RI, MS, Tent
405	1-(2-Furanyl)-1-propanone	3194-15-8	1668	1.76	901	1588	1565	2,3	RI, MS, Tent
406	5-Methyl-furfural	620-02-0	1668	1.69	938	1588	1582	1,2,3	RI, MS, STD
407	Furfuryl propionate	623-19-8	1696	1.85	802	1604	1603	3	RI, MS, STD
408	2-Methyl-benzofuran	4265-25-2	1708	2.02	905	1610	1589	1	RI, MS, Tent
409	2-Furfurylfuran	1197-40-6	1724	1.84	883	1619	1615	1,3	RI, MS, STD
410	2-Acetyl-5-methylfuran	1193-79-9	1744	1.79	945	1630	1608	1,2	RI, MS, STD
411	Ethyl 2-furoate	614-99-3	1748	1.79	937	1632	1621	1,2,3	RI, MS, STD
412	5-Ethyl-2-furaldehyde	23074-10-4	1784	1.86	763	1652	1645	1,2,3	RI, MS, STD

413	2-Furanmethanol	98-00-0	1804	1.42	954	1662	1666	1,3	RI, MS, STD
414	2-Butanoylfuran	4208-57-5	1808	1.96	733	1665	1644	2,3	RI, MS, Tent
415	Furfuryl butyrate	623-21-2	1824	2.11	915	1674	1649	1,2,3	RI, MS, STD
416	2-Furfuryl-5-methylfuran	13678-51-8	1844	2.18	849	1685	1703	1	RI, MS, Tent
417	5-Methyl-2-propionylfuran	10599-69-6	1860	2.03	841	1693	1686	2,3	RI, MS, Tent
418	Propyl 2-furoate	615-10-1	1900	1.93	847	1715	1696	1,2,3	RI, MS, Tent
419	Furfuryl pentanoate	36701-01-6	2008	2.03	834	1774	1753	1,2,3	RI, MS, STD
420	Furfuryl hexanoate	39252-02-3	2168	1.98	914	1874	1857	1,2,3	RI, MS, STD
421	2-Hexanoylfuran	14360-50-0	2184	1.85	840	1884	1872	3	RI, MS, Tent
422	Dibenzofuran	132-64-9	2656	1.96	909	2307	2308	1,2,3	RI, MS, Tent
423	5-Hydroxymethylfurfural	67-47-0	2884	1.5	834	2527	2528	2,3	RI, MS, STD
	**Nitrogen compounds**								
424	Pyridine	110-86-1	900	1.68	839	1197	1193	2,3	RI, MS, Tent
425	Trimethyl-oxazole	20662-84-4	916	1.88	860	1205	1206	2	RI, MS, Tent
426	Pyrazine	290-37-9	948	1.62	712	1220	1219	3	RI, MS, STD
427	2-Methyl pyrazine	109-08-0	1064	1.77	837	1276	1271	2	RI, MS, STD
428	2,5-Dimethyl-pyrazine	123-32-0	1176	1.92	714	1331	1333	2,3	RI, MS, STD
429	2,6-Dimethyl-pyrazine	108-50-9	1184	1.94	881	1335	1308	2	RI, MS, STD
430	Ethyl-pyrazine	13925-00-3	1196	1.91	885	1341	1344	2	RI, MS, STD
431	2,3-Dimethyl-pyrazine	5910-89-4	1224	1.98	863	1355	1377	2,3	RI, MS, STD
432	Isopropylpyrazine	9820-90-0	1232	2.04	762	1359	1385	2,3	RI, MS, Tent

433	2-Ethyl-6-methyl-pyrazine	13925-03-6	1296	2.12	899	1391	1395	2,3	RI, MS, STD
434	2-Methyl-3-isopropylpyrazine	15986-81-9	1304	2.25	746	1395	1387	2	RI, MS, Tent
435	2-Ethyl-5-methyl-pyrazine	13360-64-0	1308	2.1	745	1397	1402	2	RI, MS, STD
436	Trimethyl-pyrazine	14667-55-1	1344	2.07	858	1416	1421	1,2,3	RI, MS, STD
437	2,6-Diethyl-pyrazine	13067-27-1	1396	2.29	741	1443	1415	3	RI, MS, STD
438	3-Ethyl-2,5-dimethyl-pyrazine	13360-65-1	1420	2.26	757	1455	1441	1,2,3	RI, MS, STD
439	2,3-Dimethyl-5-ethylpyrazine	15707-34-3	1444	2.25	898	1468	1460	2,3	RI, MS, STD
440	Tetramethyl-pyrazine	1124-11-4	1476	2.21	947	1484	1462	1,2,3	RI, MS, STD
441	3,5-Diethyl-2-methyl-pyrazine	18138-05-1	1508	2.41	742	1501	1503	3	RI, MS, STD
442	2,3,5-Trimethyl-6-ethylpyrazine	17398-16-2	1544	2.39	887	1521	1491	3	RI, MS, STD
443	1H-Pyrrole	109-97-7	1548	1.35	737	1523	1513	1,2,3	RI, MS, STD
444	2-Methyl-1H-pyrrole	636-41-9	1624	1.42	887	1564	1551	3	RI, MS, Tent
445	3-Methyl-1H-pyrrole	616-43-3	1652	1.41	863	1579	1569	2,3	RI, MS, Tent
446	2-Pentyl-Pyridine	2294-76-0	1676	2.28	733	1593	1572	2	RI, MS, Tent
447	2-Formyl-1-methylpyrrole	1192-58-1	1756	1.79	838	1636	1626	2	RI, MS, Tent
448	2-Acetyl-6-methylpyridine	6940-57-4	1764	2	713	1641	1614	2	RI, MS, Tent
449	2,3-Dimethyl-5-isopentylpyrazine	18450-01-6	1812	2.73	717	1667	1680	3	RI, MS, Tent
450	Quinoline	91-22-5	2296	1.82	759	1964	1942	2	RI, MS, Tent
451	2-Acetylpyrrole	1072-83-9	2328	1.45	878	1987	2002	1,2,3	RI, MS, STD
452	2-Formylpyrrole	1003-29-8	2400	1.37	820	2046	2059	1,2	RI, MS, STD
	**Sulfides**								

453	Methanethiol	74-93-1	292	1.34	985	669	643	1,3	RI, MS, STD
454	Dimethyl sulfide	75-18-3	316	1.43	895	750	774	1	RI, MS, STD
455	Methyl thiolacetate	1534-08-3	628	1.69	814	1054	1052	1,2	RI, MS, Tent
456	Dimethyl disulfide	624-92-0	668	1.81	960	1077	1078	1,2,3	RI, MS, STD
457	S-Methyl propanethioate	5925-75-7	752	1.95	749	1122	1131	1,2,3	RI, MS, STD
458	Methyl ethyl disulfide	20333-39-5	804	2.05	736	1149	1141	2	RI, MS, Tent
459	S-Methyl ester butanethioic acid	2432-51-1	908	2.13	835	1201	1198	1,2,3	RI, MS, STD
460	Thiazole	288-47-1	1032	1.59	907	1261	1259	2,3	RI, MS, STD
461	Dimethyl trisulphide	3658-80-8	1312	2.16	966	1399	1400	1,2,3	RI, MS, STD
462	S-Methyl hexanethioate	2432-77-1	1340	2.37	895	1414	1412	1,2,3	RI, MS, Tent
463	Methyl pentyl disulfide	72437-68-4	1400	2.48	764	1445	1445	3	RI, MS, Tent
464	4,5-Dimethyl-2-isopropyl-thiazole	53498-30-9	1424	2.47	747	1457	1436	3	RI, MS, Tent
465	Ethyl 2-(methylthio)acetate	4455-13-4	1428	1.88	902	1459	1484	1	RI, MS, STD
466	Methional	3268-49-3	1448	1.72	826	1470	1480	1,2,3	RI, MS, STD
467	2-Pentyl-thiophene	4861-58-9	1448	2.51	893	1470	1452	1,2,3	RI, MS, Tent
468	Furfuryl methyl sulfide	1438-91-1	1504	1.87	913	1499	1492	1,3	RI, MS, Tent
469	4,5-Dimethyl-2-isobutylthiazole	53498-32-1	1568	2.57	709	1534	1514	3	RI, MS, Tent
470	2-(Methylthio)ethanol	5271-38-5	1576	1.5	725	1538	1520	1	RI, MS, Tent
471	Methyl propyl trisulfide	17619-36-2	1588	2.47	752	1545	1529	1	RI, MS, Tent
472	Ethyl 3-(methylthio)propionate	13327-56-5	1644	2	961	1575	1580	1,2,3	RI, MS, STD
473	2,5-Dimethyl-1,3,4-trithiolane	23654-92-4	1724	2.32	865	1619	1618	1,3	RI, MS, Tent

474	3-(Methylthio)propyl acetate	16630-55-0	1760	1.99	752	1639	1627	1	RI, MS, Tent
475	2,4,5-Trithiahexane	42474-44-2	1828	2.26	895	1676	1662	1,2,3	RI, MS, Tent
476	Methyl benzyl sulfide	766-92-7	1836	2.36	932	1680	1665	1,3	RI, MS, STD
477	3-Thiophenecarboxaldehyde	498-62-4	1868	1.77	711	1697	1687	1,2,3	RI, MS, Tent
478	2-Thiophenecarboxaldehyde	1998-3-3	1896	1.73	920	1713	1722	1,2,3	RI, MS, STD
479	Methionol	505-10-2	1916	1.56	914	1724	1721	1,2,3	RI, MS, STD
480	5-Methyl-2-formylthiophene	13679-70-4	1932	1.9	814	1733	1759	1,2,3	RI, MS, Tent
481	Dimethyl tetrasulphide	5756-24-1	1988	2.32	727	1763	1750	3	RI, MS, Tent
482	1,2,4-Trithiolane	289-16-7	2004	2	866	1772	1760	1,3	RI, MS, Tent
483	3-Acetylthiophene	1468-83-3	2044	1.75	752	1794	1772	2	RI, MS, Tent
484	2-Acetylthiophen	88-15-3	2044	1.74	717	1794	1785	3	RI, MS, STD
485	Furfuryl methyl disulfide	57500-00-2	2088	1.94	846	1822	1813	1,2,3	RI, MS, Tent
486	3-Methyl-2-thiophenecarbaldehyde	5834-16-2	2104	1.76	798	1832	1815	1,2,3	RI, MS, Tent
487	1-(2-Thienyl) propanone	13679-75-9	2144	1.8	714	1858	1840	1	RI, MS, Tent
488	Benzothiazole	95-16-9	2320	1.78	835	1981	1958	1,2,3	RI, MS, STD
489	2-Phenylthiophene	825-55-8	2476	1.76	780	2114	2124	1	RI, MS, STD
	**Phenols**								
490	2-Methoxy phenol	1990-5-1	2168	1.55	954	1874	1871	1,2,3	RI, MS, STD
491	Di-ter-butyl p-cresol	128-37-0	2232	2.24	928	1918	1910	1,2,3	RI, MS, Tent
492	2-Methoxy-4-methylphenol	93-51-6	2304	1.57	951	1969	1956	1,2,3	RI, MS, STD
493	Phenol	108-95-2	2364	1.33	967	2015	2030	1,2,3	RI, MS, STD

494	2-Methylphenol	95-48-7	2360	1.38	941	2012	1996	1,2,3	RI, MS, STD
495	2-Methoxy-4-ethylphenol	2785-89-9	2400	1.56	922	2046	2048	1,2,3	RI, MS, STD
496	p-Cresol	106-44-5	2456	1.34	957	2094	2089	1,2,3	RI, MS, STD
497	2-Methoxy-4-propylphenol	2785-87-7	2484	1.56	883	2122	2139	1,2	RI, MS, STD
498	3-Ethylphenol	620-17-7	2524	1.38	731	2163	2181	3	RI, MS, Tent
499	2-tert-Butylphenol	88-18-6	2532	1.43	729	2172	2161	3	RI, MS, Tent
500	4-Ethylphenol	123-07-9	2548	1.38	930	2188	2195	1,2,3	RI, MS, STD
501	5-Methyl-2-isopropylphenol	89-83-8	2548	1.45	844	2188	2180	1,2,3	RI, MS, Tent
502	2-Methoxy-4-vinylphenol	7786-61-0	2572	1.53	867	2214	2213	2	RI, MS, STD
503	2-Methyl-5-isopropylphenol	499-75-2	2576	1.47	836	2219	2227	1	RI, MS, Tent
504	4-Propylphenol	645-56-7	2628	1.45	737	2276	2270	1,2,3	RI, MS, Tent
505	2,6-Dimethoxyphenol	1991-10-1	2636	1.61	740	2285	2269	2,3	RI, MS, STD
506	2,4-Di-tert-butylphenol	96-76-4	2656	1.64	898	2307	2317	1,2,3	RI, MS, Tent
507	4-Ethenylphenol	2628-17-3	2756	1.44	773	2410	2425	3	RI, MS, STD
	**Terpenes**								
508	δ-3-Carene	13466-78-9	784	2.98	842	1139	1166	2	RI, MS, Tent
509	α-Limonene	138-86-3	892	2.8	913	1193	1200	1,2	RI, MS, STD
510	1,8-Cineole	470-82-6	920	2.97	862	1207	1211	1,2,3	RI, MS, Tent
511	Terpinolene	586-62-9	1124	2.69	886	1306	1280	1	RI, MS, STD
512	α-Thujone	546-80-5	1340	2.44	790	1414	1431	3	RI, MS, Tent
513	trans-Linalool oxide	34995-77-2	1460	1.99	925	1476	1483	3	RI, MS, Tent

514	cis-Linalool oxide	5989-33-3	1464	2.02	921	1478	1454	1,2,3	RI, MS, Tent
515	α-Longipinene	5989-8-2	1472	3.59	828	1483	1482	1	RI, MS, Tent
516	α-Copaene	3856-25-5	1504	3.63	831	1500	1497	3	RI, MS, Tent
517	Daucene	16661-00-0	1508	3.63	879	1502	1495	1	RI, MS, STD
518	Longicyclene	1137-12-8	1528	3.67	889	1513	1497	1	RI, MS, Tent
519	Theaspirane B	43126-21-2	1532	3.17	717	1515	1522	1	RI, MS, Tent
520	Camphor	76-22-2	1532	3.87	758	1515	1540	1,2,3	RI, MS, STD
521	(-)-Camphor	464-48-2	1564	2.41	949	1532	1532	1	RI, MS, Tent
522	Vitispirane	65416-59-3	1576	2.95	853	1539	1527	1,3	RI, MS, Tent
523	α-Gurjunene	489-40-7	1580	3.77	903	1541	1529	1,3	RI, MS, Tent
524	Linalool	78-70-6	1588	1.81	947	1545	1552	1,2,3	RI, MS, STD
525	Theaspirane	36431-72-8	1600	3.11	838	1552	1523	1	RI, MS, Tent
526	α-Cedrene	469-61-4	1628	3.74	877	1568	1571	2	RI, MS, STD
527	Carvomenthone	499-70-7	1628	2.46	753	1567	1552	1,3	RI, MS, Tent
528	β-Funebrene	79120-98-2	1636	3.7	860	1572	1588	2,3	RI, MS, Tent
529	Junipene	475-20-7	1656	3.61	926	1583	1583	1	RI, MS, Tent
530	D-Fenchyl alcohol	1632-73-1	1664	1.87	942	1586	1588	1,3	RI, MS, Tent
531	α-trans-Bergamotene	13474-59-4	1672	3.35	903	1591	1583	1	RI, MS, Tent
532	α-Guaiene	3691-12-1	1684	3.48	860	1598	1598	1	RI, MS, Tent
533	β-Elemene	515-13-9	1684	3.04	908	1598	1586	1	RI, MS, Tent
534	Calarene	17334-55-3	1692	3.62	916	1602	1604	1,2,3	RI, MS, STD

535	trans-Caryophyllene	87-44-5	1700	3.43	949	1607	1581	1,3	RI, MS, STD
536	Terpinen-4-ol	562-74-3	1704	2.09	940	1608	1628	1,2,3	RI, MS, STD
537	Isophorone	78-59-1	1708	2.07	920	1610	1600	1,2,3	RI, MS, STD
538	trans-Edulan	41678-29-9	1720	2.8	748	1617	1620	3	RI, MS, Tent
539	β-Terpineol	138-87-4	1748	1.91	862	1632	1616	1	RI, MS, Tent
540	β-Cyclocitral	432-25-7	1748	2.4	824	1632	1613	1,3	RI, MS, STD
541	α-Patchoulene	560-32-7	1776	3.86	819	1648	1640	1	RI, MS, Tent
542	Alloaromadendrene	25246-27-9	1788	3.88	884	1655	1644	1,3	RI, MS, Tent
543	β-Barbatene	72346-55-5	1800	3.84	746	1662	1667	1	RI, MS, Tent
544	γ-Gurjunene	22567-17-5	1804	3.93	919	1664	1674	1	RI, MS, Tent
545	Isoborneol	124-76-5	1820	2.03	803	1671	1672	1	RI, MS, Tent
546	α-Humulene	6753-98-6	1832	3.78	919	1679	1680	1,3	RI, MS, Tent
547	L-Borneol	464-45-9	1852	2.05	730	1689	1675	1	RI, MS, Tent
548	α-Terpineol	98-55-5	1872	2.02	958	1700	1700	1,2	RI, MS, STD
549	γ-Amorphene	6980-46-7	1864	3.68	895	1696	1724	1	RI, MS, Tent
550	Ledene	21747-46-6	1880	3.68	902	1705	1701	1	RI, MS, Tent
551	trans-Borneol	507-70-0	1880	1.95	924	1704	1679	1,2,3	RI, MS, Tent
552	β-Chamigrene	18431-82-8	1900	3.66	864	1716	1702	1	RI, MS, Tent
553	Valencene	4630-7-3	1928	3.44	899	1731	1726	1	RI, MS, Tent
554	α-bisabolene	25532-79-0	1936	3.18	878	1735	1720	1,2,3	RI, MS, STD
555	Germacrene A	28387-44-2	1956	3.37	839	1747	1743	3	RI, MS, Tent

556	α-Chamigrene	19912-83-5	1960	3.46	851	1749	1753	1,3	RI, MS, Tent
557	δ-Cadinene	483-76-1	1988	3.25	932	1764	1753	1,2,3	RI, MS, STD
558	β-Citronellol	106-22-9	1992	1.77	889	1765	1771	1	RI, MS, STD
559	7 epi-a-Selinene	123123-37-5	2008	3.26	873	1775	1772	1	RI, MS, Tent
560	α-Curcumene	644-30-4	2016	2.79	881	1779	1788	1,2	RI, MS, Tent
561	Nerol	106-25-2	2072	1.7	845	1811	1821	3	RI, MS, Tent
562	Isogeraniol	5944-20-7	2096	1.69	832	1827	1818	3	RI, MS, Tent
563	β-Damascenone	23726-93-4	2104	2.26	910	1832	1827	1,3	RI, MS, STD
564	Dihydro-β-ionone	17283-81-7	2124	2.36	835	1845	1854	1,2,3	RI, MS, Tent
565	L-calamenene	483-77-2	2124	2.81	946	1846	1838	1,2,3	RI, MS, STD
566	Geraniol	106-24-1	2132	1.7	872	1850	1851	1,3	RI, MS, STD
567	trans-Geranylacetone	3796-70-1	2148	2.19	877	1861	1862	1,2	RI, MS, STD
568	Geosmin	19700-21-1	2148	2.32	902	1861	1858	1,2,3	RI, MS, STD
569	α-Ionone	127-41-3	2156	2.2	846	1866	1866	1	RI, MS, STD
570	α-Dehydro-himachalene	78204-62-3	2184	2.61	836	1885	1882	1	RI, MS, Tent
571	α-Calacorene	21391-99-1	2248	2.53	898	1930	1904	1,2,3	RI, MS, Tent
572	Palustrol	5986-49-2	2264	2.46	899	1941	1938	1,2,3	RI, MS, Tent
573	trans-β-Ionone	79-77-6	2280	2.15	854	1952	1953	1,2,3	RI, MS, STD
574	cis-Jasmone	488-10-8	2292	1.99	859	1961	1955	3	RI, MS, STD
575	β-Caryophyllene oxide	1139-30-6	2296	2.17	792	1964	1990	1,2,3	RI, MS, Tent
576	D-Nerolidol	142-50-7	2388	1.84	921	2036	2010	1,3	RI, MS, Tent

577	E-Nerolidol	40716-66-3	2392	1.82	926	2040	2054	2	RI, MS, Tent
578	Epicubenol	19912-67-5	2436	2.07	765	2077	2078	1	RI, MS, Tent
579	α-Corocalene	20129-39-9	2436	2.15	863	2077	2083	1	RI, MS, Tent
580	Cubenol	21284-22-0	2436	2.07	787	2077	2071	2	RI, MS, Tent
581	6-Isocedrol	19903-73-2	2496	1.95	894	2135	2162	1	RI, MS, Tent
582	α-Cedrol	77-53-2	2496	1.95	877	2135	2127	2,3	RI, MS, Tent
583	β-Bisabolol	15352-77-9	2520	1.82	728	2160	2151	3	RI, MS, Tent
584	Torreyol	19435-97-3	2556	1.92	815	2197	2197	2,3	RI, MS, Tent
585	α-Cadinol	481-34-5	2556	1.92	810	2197	2217	1,3	RI, MS, STD
586	α-Eudesmol	473-16-5	2592	1.98	719	2237	2223	3	RI, MS, Tent
587	β-Eudesmol	473-15-4	2600	2	821	2246	2246	3	RI, MS, Tent
588	Farnesol	4602-84-0	2700	1.95	846	2353	2351	2	RI, MS, Tent
589	9H-Fluorene	86-73-7	2732	2.16	907	2386	2374	2,3	RI, MS, Tent
	**Lactones**								
590	γ-Pentalactone	108-29-2	1744	1.71	922	1630	1648	1,3	RI, MS, STD
591	Butyrolactone	96-48-0	1784	1.68	955	1652	1643	1,2,3	RI, MS, STD
592	γ-Vinyl-γ-valerolactone	1073-11-6	1848	1.87	917	1687	1679	2	RI, MS, Tent
593	γ-Caprolactone	695-06-7	1916	1.83	784	1724	1703	1	RI, MS, STD
594	α-Methyl-γ-crotonolactone	22122-36-7	1948	1.65	940	1741	1726	2,3	RI, MS, Tent
595	3,4-Dimethyl-2,5-furandione	766-39-2	1968	1.68	777	1752	1764	2	RI, MS, STD
596	γ-Crotonolactone	497-23-4	2016	1.52	942	1778	1787	2,3	RI, MS, STD

597	δ-Hexalactone	823-22-3	2080	1.76	937	1816	1818	1,2,3	RI, MS, Tent
598	γ-Octalactone	104-50-7	2264	1.77	956	1941	1924	2,3	RI, MS, Tent
599	δ-Octalactone	698-76-0	2336	1.79	772	1993	1999	2,3	RI, MS, Tent
600	γ-Nonalactone	104-61-0	2408	1.7	937	2053	2044	1	RI, MS, STD
601	γ-Carboethoxy-γ-butyrolactone	1126-51-8	2512	1.49	727	2151	2168	3	RI, MS, Tent
602	γ-Decalactone	706-14-9	2532	1.68	905	2172	2149	1,2,3	RI, MS, STD
603	δ-Decalactone	705-86-2	2580	1.79	801	2224	2220	2,3	RI, MS, Tent
604	γ-Dodecalactone	2305-5-7	2748	2.07	885	2403	2381	2	RI, MS, STD
605	γ-6-(Z)-Dodecenolactone	18679-18-0	2772	2.08	874	2426	2425	1,2,3	RI, MS, Tent
606	δ-Heptyl-δ-valerolactone	713-95-1	2808	2.22	814	2460	2458	3	RI, MS, Tent

[a] RT1: retention time on the primary column.

[b] RT2: retention time on the secondary column.

[c] LRIcal: calculated linear retention indices.

[d] LRIlit: literature linear retention indices obtained from the NIST library (https://webbook.nist.gov/chemistry/).

[e] Origin: compounds identified by “1” HS-SPME-GC×GC-TOFMS, “2” SPE-GC×GC-TOFMS, “3” SBSE-GC×GC-TOFMS.

[f] Identification: tentative identification (Tent.) based on retention indices (RI) and mass spectra (MS), positive identification based on retention times of authentic standards (STD).

Table S2. Peak area of 606 volatile compounds identified in Chinese herbaceous aroma-type Baijiu.

NO.	Compounds	Area[a] & RSD					
	**Esters**	SPME	RSD	SPE	RSD	SBSE	RSD
1	Methyl acetate	209845	16788	-	-	-	-
2	Ethyl acetate	59168668	5325180	-	-	68874663	6198720
3	Ethyl propanoate	3784650	378465	17450771	4498021	9193378	919338
4	Ethyl 2-methyl propanoate	918006934	100980763	2457528	270328	1877440	206518
5	Propyl acetate	111253363	6675202	162737695	9764262	351467	21088
6	2-Butyl acetate	42866784	2657741	182922837	11341216	324213476	20101236
7	Methyl butanoate	-	-	19905183	1273932	-	-
8	Ethyl acrylate	-	-	1186052	78279	148206	9782
9	Methyl 2-methylbutyrate	-	-	764041	51955	-	-
10	Isobutyl acetate	333336983	23673589	204341025	14303872	2970513	207936
11	Methyl isovalerate	-	-	2109554	151888	-	-
12	Allyl acetate	-	-	973004	72002	-	-
13	Ethyl butanoate	128228	9745	33052	2512	3573013	271549
14	Propyl propionate	-	-	61632259	4807316	362715	28292
15	Ethyl 2-methylbutanoate	20731123	1658490	200003071	16000246	5727750	458220
16	Butyl acetate	75083897	6156880	52477241	4303134	17550135	1439111
17	Ethyl 3-methylbutanoate	65338655	5488447	47136	3959	398409	33466

18	Methyl pentanoate	45108	3879	11025200	948167	173008	14879
19	Isobutyl isobutyrate	39665864	3490596	4998456	439864	-	-
20	Isobutyl propanoate	44623882	3168296	58759728	4171941	141172471	10023245
21	Propyl butyrate	1026598791	73915113	263709173	18987060	8696564	626153
22	3-Methylbutyl acetate	698234323	50971106	45459692	3318558	2639292	192668
23	sec-Butyl butyrate	38128056	2821476	3941376	291662	155036	11473
24	Propyl 2-methylbutanoate	2221291	166597	1652170	123913	-	-
25	Butyl propionate	-	-	30071082	2285402	-	-
26	1-Methylpropyl 2-methylbutanoate	65019	5006	-	-	-	-
27	Butyl isobutyrate	-	-	6589584	513988	-	-
28	Propyl isovalerate	68823629	5437067	20808719	1643889	341961950	27014994
29	Isobutyl isovalerate	-	-	3405933	272475	-	-
30	Ethyl valerate	245792747	19909213	4575481	370614	1307822129	105933592
31	Ethyl 2-butenoate	-	-	2937740	240895	2852061	233869
32	Isobutyl butyrate	365845594	30365184	83339362	6917167	-	-
33	Isobutyl 2-methylbutanoate	-	-	316795	26611	-	-
34	Allyl butanoate	-	-	1744188	148256	-	-
35	Ethyl 3-methylvalerate	-	-	547692	47102	-	-
36	Isopentyl isobutyrate	337856	17569	-	-	-	-
37	Methyl hexanoate	174038078	9224018	118498406	6280416	1322152821	70074100
38	Amyl acetate	16859297	910402	29222445	1578012	2225471	120175

39	Ethyl 4-methylvalerate	9655882	531074	22171489	1219432	1391205	76516
40	2-Pentyl butanoate	-	-	5665093	317245	47506	2660
41	Isoamyl propionate	-	-	-	-	191257	10902
42	Butyl butyrate	430724	24982	27379849	1588031	-	-
43	Propyl valerate	212923538	12562489	44766053	2641197	-	-
44	2-Butyl-n-valerate	-	-	967128	58028	-	-
45	Pentyl isobutyrate	-	-	1091176	66562	-	-
46	Butyl isovalerate	-	-	6098001	378076	-	-
47	Isobutyl valerate	-	-	19448294	1225243	-	-
48	Ethyl hexanoate	22830546	1461155	1494043	95619	5993132	383560
49	1-Methylhexyl acetate	-	-	59330781	3856501	-	-
50	Isopentyl butanoate	618103	40795	-	-	-	-
51	Hexyl acetate	68508582	4590075	531720	35625	18086390	1211788
52	Isopentyl 2-methylbutanoate	38758895	2635605	3120359	212184	10477600	712477
53	Methyl heptanoate	6751017	465820	-	-	27126051	1871698
54	Isopentyl isovalerate	55236687	3866568	5571361	389995	-	-
55	Ethyl (E)-3-hexenoate	27075186	1922338	5358343	380442	8277019	587668
56	Ethyl lactate	-	-	-	-	165759	11935
57	Propyl hexanoate	7033988	513481	385008	28106	989363	72223
58	(Z)-3-hexen-1-yl acetate	-	-	-	-	243654	18030
59	Ethyl 2-hydroxypropanoate	-	-	34549491	2591212	111701097	8377582

60	Amyl 2-methylbutyrate	11805063	897185	649331	49349	2063856	156853
61	Ethyl β-ethoxypropionate	2725870	209892	5431315	418211	13428170	1033969
62	Ethyl 2-hexenoate	-	-	111039	8661	-	-
63	Ethyl heptanoate	184321276	14561381	71124	5619	487704	38529
64	Hexyl propanoate	194804	15584	12425370	994030	46322051	3705764
65	Hexyl isobutyrate	33933970	2748652	3507824	284134	137982	11177
66	Amyl isovalerate	392115	32153	421108	34531	1744349	143037
67	Isobutyl hexanoate	225798	18741	946122	78528	80042538	6643531
68	Isoamyl valerate	228587255	19201329	4255197	357437	1216274580	102167065
69	2-Propenyl hexanoate	17791736	1512298	2130673	181107	-	-
70	Heptyl acetate	9002080	774179	581658	50023	5743723	493960
71	Ethyl Z-4-heptenoate	827990	72035	-	-	689046	59947
72	Methyl octanoate	26011337	2288998	1748974	153910	10362063	911862
73	2-Heptyl butanoate	-	-	753328	67046	335322	29844
74	Cyclopentyl butyrate	674435	60699	-	-	-	-
75	Ethyl 2-hydroxybutyrate	4116176	374572	2355684	214367	-	-
76	Ethyl 3-hydroxy-3-methylbutyrate	-	-	371026	16696	-	-
77	Butyl hexanoate	16385742	753744	331730	15260	2405517	110654
78	Propyl heptanoate	-	-	18585654	873526	38562790	1812451
79	Hexyl butanoate	21685118	1040886	61984163	2975240	66390672	3186752
80	Isopropyl octanoate	-	-	66948	3280	242722583	11893407

81	Propyl lactate	33718989	1685949	765638	38282	56858739	2842937
82	Ethyl 2-hydroxy-3-methylbutyrate	161855413	8254626	110203517	5620379	1229602124	62709708
83	Hexyl 2-methylbutyrate	86473112	4496602	4699073	244352	5453284	283571
84	Ethyl cyclohexanoate	5489867	290963	531406	28165	7426470	393603
85	Ethyl octoate	109494	5913	279857	15112	867700	46856
86	Heptyl propionate	227071	12489	-	-	320814	17645
87	Hexyl isopentanoate	46751862	2618104	2794974	156519	5527379	309533
88	Isoamyl hexanoate	-	-	97995604	5585749	4752415	270888
89	Isobutyl heptanoate	769934	44656	-	-	-	-
90	Isobutyl lactate	30530096	1801276	91373534	5391039	54711341	3227969
91	2-Methylbutyl hexanoate	615433577	36926015	6605472	396328	-	-
92	2-Ethylhexyl 2-propenoate	913067	55697	-	-	-	-
93	Ethyl 7-octenoate	3448112	213783	300596	18637	829191	51410
94	Ethyl diethoxyacetate	1586051	99921	1783499	112360	5430091	342096
95	Methyl nonanoate	519358	33239	-	-	-	-
96	Pentyl hexanoate	174229	11325	82771	5380	2312783	150331
97	Ethyl 3-hydroxybutyrate	2290060	151144	1234681	81489	-	-
98	Heptyl butanoate	55689	3731	-	-	-	-
99	Butyl 2-hydroxypropanoate	100733	6850	37243837	2532581	22934103	1559519
100	Propyl octanoate	182586165	12598445	27064859	1867475	7176582	495184
101	Heptyl 2-methylbutanoate	100733	7051	-	-	-	-

102	Ethyl nonanoate	33411750	2372234	994260	70592	18726	1330
103	Octyl propanoate	1120077	80646	-	-	-	-
104	Ethyl 2-hydroxy-4-methylpentanoate	31504922	2299859	-	-	29471013	2151384
105	Ethyl E-2-octenoate	-	-	-	-	82518	6106
106	Isopentyl heptanoate	-	-	-	-	2474514	185589
107	Nonyl acetate	671575	51040	-	-	-	-
108	Ethyl 3-acetoxybutanoate	-	-	-	-	388668	29927
109	Diethyl propanedioate	241541	18840	684292	53375	672087	52423
110	2-Heptyl hexanoate	286499	22633	7422111	586347	1348787	106554
111	2-Camphanol acetate	-	-	-	-	328610	26289
112	3-methyl-2-butenyl hexanoate	14111491	1270034	-	-	-	-
113	Methyl decanoate	2385806	238581	-	-	159572	15957
114	Hexyl hexanoate	34963103	3845941	8929847	982283	97008	10671
115	Ethyl levulate	807528	48452	4758751	285525	1410544	84633
116	Octyl butanoate	11214426	695294	283010	17547	-	-
117	Butyl octanoate	465981	29823	4864536	311330	-	-
118	Octyl 2-methylbutanoate	331109	21853	-	-	-	-
119	Methyl benzoate	1691868	115047	1031799	70162	5686049	386651
120	Ethyl decanoate	1013429	70940	115988506	8119195	132315833	9262108
121	Ethyl methyl butanedioate	-	-	472109	33992	266539	19191
122	Diethyl fumarate	525583	38893	285386	21119	1752732	129702

123	Isoamyl octanoate	2187233	166230	200448	15234	225222	17117
124	Ethyl trans-4-decenoate	130405	10172	-	-	235335	18356
125	Isopropyl benzoate	170542	13643	-	-	188776	15102
126	Diethyl succinate	17391	1426	39084	3205	262771	21547
127	Ethyl benzoate	27999659	2351971	49832401	4185922	46338499	3892434
128	Ethyl 3-hydroxyhexanoate	-	-	1656812	142486	-	-
129	Ethyl cis-4-decenoate	703092	61872	-	-	-	-
130	Ethyl 9-decenoate	177496	12602	-	-	-	-
131	α-Terpineol acetate	333602	24019	-	-	-	-
132	α-Phenylethyl acetate	-	-	-	-	124904	9118
133	Propyl decanoate	-	-	1457006	107818	300791	22259
134	Benzyl acetate	959295	71947	618630	46397	3665894	274942
135	Ethyl undecanoate	2817758	214150	1076617	81823	148788	11308
136	Butyl butyrolactate	2182058	168018	-	-	-	-
137	Ethyl trans-2-decenoate	584718	45608	-	-	-	-
138	Propyl benzoate	893562	70591	-	-	-	-
139	Methyl phenylacetate	1348511	107881	1117720	89418	2123240	169859
140	Ethyl glutarate	-	-	323376	26193	1843550	149328
141	Benzyl isobutanoate	559967	45917	-	-	-	-
142	Methyl salicylate	-	-	-	-	413143	34291
143	Ethyl phenylacetate	105739506	8882119	-	-	119654159	10050949

144	Isobutyl benzoate	1428704	121440	316283	26884	1281367	108916
145	Benzyl propionate	1231754	105931	-	-	1139546	98001
146	Hexyl octanoate	137791	7165	77817	4046	233109	12122
147	β-Phenethyl acetate	80687954	4276462	53982639	2861080	81786	4335
148	Ethyl dodecanoate	128489127	6938413	55969973	3022379	47825663	2582586
149	Methyl benzenepropanoate	1031282	56721	-	-	4122987	226764
150	n-Butyl benzoate	671327	37594	-	-	-	-
151	Benzyl butanoate	-	-	-	-	5626704	320722
152	β-Phenylethyl isobutyrate	-	-	-	-	16808684	974904
153	Ethyl 3-phenylpropionate	424791	25063	192348	11349	240895	14213
154	Propyl dodecanoate	721987	43319	-	-	473280	28397
155	Isoamyl benzoate	1383932	84420	-	-	20395186	1244106
156	Benzenepropyl acetate	664755	41215	-	-	3133000	194246
157	Butyl phenylacetate	23011779	1449742	4747252	299077	21888194	1378956
158	2-Phenylethyl butanoate	14286373	914328	-	-	161154384	10313881
159	Phenethyl 2-methylbutyrate	14111491	917247	-	-	-	-
160	Phenethyl isovalerate	17893562	1180975	1754258	115781	3700843	244256
161	Ethyl tetradecanoate	-	-	13236833	886868	37477711	2511007
162	Diethyl dl-malate	-	-	198536	13500	-	-
163	Triacetyl glycerol	-	-	5959668	411217	-	-
164	Diethyl octanedioate	-	-	66750	4673	238001	16660

165	Ethyl cinnamate	1388781	98603	785996	55806	69446	4931
166	Ethyl pentadecanoate	828562	59656	221805	15970	530270	38179
167	Hexyl phenylacetate	1394759	101817	853874	62333	-	-
168	Methyl hexadecanoate	-	-	-	-	37582	2781
169	Ethyl hexadecanoate	222310	16673	125467	9410	118843	8913
170	Ethyl hexadec-9-enoate	-	-	1589937	120835	108800	8269
171	Hexyl dihydrocinnamate	1037441	79883	-	-	-	-
172	Ethyl heptadecanoate	-	-	151895	11848	1881888	146787
173	Propyl hexadecanoate	-	-	-	-	14082061	1112483
174	Ethyl hydrogen succinate	3461152	276892	11881839	950547	-	-
175	Butyl hexadecanoate	-	-	-	-	1552624	125763
176	Ethyl octadecanoate	-	-	-	-	103101	8454
177	Ethyl cis-9-octadecenoate	-	-	6105539	506760	4777822	396559
178	Ethyl linoleate	-	-	110293	9265	68036234	5715044
179	Ethyl vanillate	-	-	421139	35797	31571	2684
	**Alcohols**						
180	2-Propanol	2271224	197596	-	-	-	-
181	2-Butanol	583034821	51307064	3672614	323190	323209	28442
182	1-Propanol	20596081	1833051	-	-	18527613	1648958
183	2-Methyl-3-buten-2-ol	-	-	1297191	116747	-	-
184	2-Methyl-1-propanol	378543	34447	86170912	7841553	1996208	181655

185	3-Pentanol	-	-	569797	25641	-	-
186	2-Pentanol	-	-	241694866	11117964	65728928	3023531
187	1-Butanol	1359727	63907	104671	4920	822950	38679
188	2-Methyl-3-pentanol	288043	13826	-	-	-	-
189	3-Penten-2-ol	-	-	53944478	2643279	-	-
190	3-Hexanol	-	-	346560	17328	-	-
191	2-Methylbutanol	176996	9027	4448697	226884	-	-
192	3-Methyl-1-butanol	398843	20740	130762190	6799634	2979206	154919
193	2-Hexanol	129307189	6853281	51353	2722	1316598	69780
194	3-Methyl-3-buten-1-ol	1505884	81318	2778742	150052	-	-
195	1-Pentanol	39083	2150	115359885	6344794	98573474	5421541
196	4-Heptanol	1666805	93341	527195	29523	1843916	103259
197	2-(Z)-Pentenol	-	-	342210	19506	-	-
198	(S)-2-Heptanol	-	-	-	-	11853581	687508
199	2-Ethyl-1-butanol	-	-	246134	14522	131727	7772
200	2-Methyl-1-pentanol	-	-	-	-	3642435	218546
201	Cyclopentanol	27937633	1704196	1912939	116689	-	-
202	Prenol	-	-	2969819	184129	183856	11399
203	4-Methyl-1-pentanol	161749	10190	-	-	43598998	2746737
204	2-Heptanol	291653656	18665834	131421701	8410989	23286380	1490328
205	3-Methyl-1-pentanol	6257938	406766	6714062	436414	3159860	205391

206	3-Methyl-cyclopentanol	-	-	701948	46329	-	-
207	1-Hexanol	367879	24648	543185488	36393428	33203	2225
208	3-Hexen-1-ol	908854	61802	-	-	-	-
209	5-Methyl-2-heptanol	-	-	-	-	1991722	137429
210	3-Ethoxy-1-propanol	3825144	267760	989825	69288	-	-
211	4-Octanol	1015434	72096	-	-	1020168	72432
212	4-Methyl-3-penten-1-ol	-	-	3270807	235498	269921	19434
213	3-Octanol	270631	19756	2964644	216419	698702	51005
214	Cyclohexanol	-	-	158772	11749	-	-
215	2-Butoxy-ethanol	190326	14274	332527	24940	119544	8966
216	cis-2-Hexenol	-	-	-	-	102435	7785
217	3-Methyl-1-hexanol	2892553	222727	-	-	410009	31571
218	5-Hexen-1-ol	1314246	102511	-	-	-	-
219	4-Methyl-1-hexanol	57736	4561	645625	51004	3475131	274535
220	1-Octen-3-ol	82903057	6632245	21348616	1707889	4899801	391984
221	1-HeptanoI	2039465	183552	62203	5598	2592952	233366
222	6-Methyl-5-hepten-2-ol	1638885	163889	105675	10568	2102998	210300
223	4-Nonanol	916313	100794	-	-	1203090	132340
224	2-Ethyl-1-hexanol	337197	20232	460112	27607	124405	7464
225	4-Hepten-1-ol	206160	12782	180873	11214	-	-
226	(E)-2-Hepten-1-ol	2029479	129887	365999	23424	2904321	185877

227	2-Nonanol	2245668	148214	2406294	158815	622718	41099
228	1-Octanol	245294	16680	37521500	2551462	105159	7151
229	p-Menthan-8-ol	-	-	-	-	717467	50223
230	5-Decanol	127250	9162	-	-	-	-
231	[S,S]-2,3-Butanediol	921584	68197	-	-	-	-
232	(Z)-3-Octen-1-ol	-	-	-	-	617132	46902
233	(Z)-5-Octen-1-ol	113666	8866	-	-	-	-
234	(E)-2-Octen-1-ol	6109714	488777	108093	8647	-	-
235	2-Octen-1-ol	-	-	-	-	193739	15887
236	2,6-Dimethyl-5-hepten-1-ol	171103	14373	-	-	-	-
237	1-Nonanol	442972	38096	5367797	461631	143401	12332
238	6-Undecanol	445767	39227	-	-	-	-
239	Undecan-4-ol	24777	1759	-	-	-	-
240	(Z)-3-Nonen-1-ol	-	-	896486	64547	5337680	384313
241	(E)-2-Nonen-1-ol	460125	33589	-	-	327542	23911
242	2-Undecanol	154185	11410	-	-	381224	28211
243	cis-6-Nonen-1-ol	298411	22381	-	-	552390	41429
244	1-Decanol	22844	1736	349969	26598	95192	7235
245	1-Methyl-1-phenylethanol	-	-	215158	16567	82791	6375
246	(Z)-4-Decen-1-ol	369530	28823	-	-	-	-
247	(Z)-3-Decen-1-ol	-	-	-	-	1250174	98764

248	α-Phenylethyl alcohol	-	-	2424960	193997	1082704	86616
249	2-Decen-1-ol	236581	19163	-	-	-	-
250	p-Cymen-8-ol	980273	80382	289013	23699	167061	13699
251	1-Undecanol	354617	29433	-	-	-	-
252	Benzenemethanol	357549	30034	13327424	1119504	2848439	239269
253	α-Ethylbenzyl alcohol	-	-	-	-	236581	20109
254	trans-2-Undecen-1-ol	177536	15268	-	-	-	-
255	Phenylethyl Alcohol	15679146	815316	27176873	1413197	2069688	107624
256	6,10-Dimethyl-5,9-undecadien-2-ol	76134	4035	-	-	-	-
257	1-Dodecanol	1719950	92877	-	-	-	-
258	Diethylene glycol	128277	7055	230581	12682	87211	4797
259	Benzenepropanol	-	-	3970084	222325	-	-
260	1-Hexadecanol	111566	6359	-	-	-	-
	**Acids**						
261	Acetic acid	34047	2009	1844919	108850	59769	3526
262	Formic acid	18534201	1112052	46316759	2779006	242512	14551
263	Propanoic acid	21232408	1295177	1389459	84757	5573612	339990
264	2-Methyl-propanoic acid	114514	7100	20391577	1264278	38334	2377
265	Butanoic acid	80549525	5074620	238144	15003	415345	26167
266	2-Methyl butyric acid	-	-	146633904	9384570	-	-
267	Pentanoic acid	1210504	78683	870073	56555	102497	6662

268	2-Methyl-pentanoic acid	9393528	619973	17327617	1143623	19652412	1297059
269	4-Methyl-pentanoic acid	2034790	136331	5906270	395720	1689525	113198
270	Hexanoic acid	99437	6762	192133007	13065044	946924652	64390876
271	5-Methyl-hexanoic acid	-	-	3935137	271524	6769640	467105
272	2-Ethyl-hexanoic acid	502029	35142	417142	29200	371061	25974
273	Heptanoic acid	2974207	211169	634488	45049	39870952	2830838
274	(E)-2-Hexenoic acid	1115221	80296	-	-	-	-
275	Octanoic acid	38644	2821	51017	3724	193672	14138
276	(E,E)-2,4-Hexadienoic acid	74202	5491	-	-	-	-
277	Nonanoic acid	3585653	268924	6230753	467306	117114	8784
278	Decanoic acid	7805237	593198	35230	2677	211400	16066
279	Benzoic acid	1203777	92691	17849975	1374448	28129719	2165988
280	Benzeneacetic acid	52428	4089	-	-	16367863	1276693
281	Tetradecanoic acid	253125	19997	178501	14102	29876	2360
282	Hexadecanoic acid	-	-	-	-	95772	7662
283	9-Hexadecenoic acid	-	-	-	-	671356	54380
	**Aldehydes & ketones**						
284	Acetaldehyde	181356316	15052574	-	-	392585	32585
285	Propanal	49319	4143	-	-	-	-
286	2-Propanone	3285152	279238	-	-	-	-
287	2-Methyl-propanal	18324322	1575892	-	-	-	-

288	2-Propenal	-	-	-	-	371057	32282
289	Butanal	1474142	129724	-	-	-	-
290	1,1-Diethoxy-ethane	101685106	9049974	-	-	33992454	3025328
291	3-Methyl-butanal	271504774	24435430	-	-	45512799	4096152
292	3-Buten-2-one	-	-	-	-	180955	16467
293	2,4,5-Trimethyl-1,3-dioxolane	25403063	1143138	-	-	47228337	2125275
294	1,1-Diethoxy-propane	120547	5545	-	-	213016	9799
295	2,3-Butanedione	-	-	-	-	5110070	240173
296	1,1-Diethoxy-2-methyl-propane	18389607	882701	16550440	794421	188405523	9043465
297	2-Pentanone	41327468	2025046	46251023	2266300	-	-
298	Pentanal	32262434	1613122	178348	8917	-	-
299	2-Methyl-1-buten-3-one	-	-	2138214	109049	88643	4521
300	4-Methyl-2-pentanone	-	-	58611	3048	-	-
301	1,1-Diethoxy-butane	-	-	2284077	121056	-	-
302	2-Butenal	-	-	1052195	56819	928422	50135
303	1,1-Diethoxy-3-methyl-butane	8979970	493898	20619484	1134072	1293820	71160
304	2-Hexanone	-	-	172435	9656	-	-
305	2,4,6-Trimethyl-1,3,5-trioxane	-	-	29408136	1676264	2732906	155776
306	Hexanal	128266988	7439485	56286076	3264592	99121793	5749064
307	2-Methyl-2-butenal	1006218	59367	125934	7430	-	-
308	E-2-Methyl-2-butenal	-	-	-	-	1024612	61477

309	4-Heptanone	-	-	91812	5601	-	-
310	(E)-3-Penten-2-one	-	-	1152885	71479	-	-
311	4-Methyl-3-penten-2-one	-	-	688612	43383	-	-
312	5-Methyl-2-hexanone	-	-	404764	25905	-	-
313	2-Methyl-2-pentenal	-	-	175533	11410	-	-
314	Acetyl valeryl	1239890	81833	18385474	1213441	28973611	1912258
315	Heptanal	8068689	540602	602907	40395	100994258	6766615
316	2-Heptanone	232875	15836	164221	11167	27486	1869
317	Cyclopentanone	952142	65698	914785	63120	1092964	75415
318	3-Ethoxy-propanal	579862	40590	-	-	-	-
319	2-Methyl-cyclopentanone	-	-	164981	11714	-	-
320	1,1-Diethoxy-hexane	-	-	324866	23390	1268822	91355
321	1-(1-Ethoxyethoxy)-hexane	8703158	635331	3354472	244876	-	-
322	3-Hydroxy-2-butanone	5154540	381436	-	-	2001084	148080
323	1,1,3-Triethoxy-dutane	-	-	23047307	1728548	-	-
324	Cyclohexanone	-	-	3822334	290497	-	-
325	1,1,3-Triethoxy-propane	-	-	32531620	2504935	24029720	1850288
326	2-Octanone	20852174	1626470	4395146	342821	40555876	3163358
327	Octanal	17740693	1401515	1770799	139893	19938177	1575116
328	4-Nonanone	-	-	806858	64549	-	-
329	1,1-Diethoxy-heptane	-	-	373778	33640	-	-

330	(E)-2-Heptenal	-	-	502188	50219	-	-
331	2,2,6-Trimethyl-cyclohexanone	-	-	-	-	4387932	482673
332	Ethyl orthoformate	163487	9809	-	-	-	-
333	(Z)-2-Heptenal	795305	49309	-	-	984253	61024
334	2-Ethyl-2-hexenal	-	-	-	-	1744476	111646
335	6-Methyl-5-heptene-2-one	672711	44399	629434	41543	4644721	306552
336	2-Nonanone	58785486	3997413	6522811	443551	-	-
337	2-Cyclopenten-1-one	-	-	2922533	204577	442202	30954
338	2-Isopropyl-5-methyl-2-hexenal	2215272	159500	-	-	228183	16429
339	2-Methyl-2-cyclopenten-1-one	-	-	163606	12107	205959	15241
340	Nonanal	400530	30440	684113	51993	10576477	803812
341	3-Octen-2-one	2805109	218799	-	-	7138085	556771
342	1,1-Diethoxy-octane	59652	4772	-	-	-	-
343	2-Octenal	3062580	251132	644961	52887	-	-
344	(E)-2-Octenal	-	-	-	-	138067	11598
345	2-Nonen-4-one	1074678	92422	-	-	-	-
346	2-Decanone	277188	24393	561182	49384	-	-
347	Decanal	3529810	250617	135833	9644	821374	58318
348	3-Nonen-2-one	3146920	226578	54808	3946	524472	37762
349	1,1-Diethoxy-nonane	63989906	4671263	377189	27535	9386775	685235
350	6-Undecanone	836137	61874	-	-	-	-

351	Benzaldehyde	117646	8823	56163	4212	5899766	442482
352	2,3-Dimethyl-2-cyclopentenone	-	-	84347	6410	-	-
353	3-Undecanone	1991440	153341	-	-	-	-
354	3,5-(E,E)-Octadien-2-one	114005	8892	-	-	-	-
355	(E,Z)-2,6-Nonadienal	254826	20131	-	-	408843	32299
356	2-Undecanone	30253439	2420275	1972097	157768	3471343	277707
357	(3E)-6-Methyl-3,5-heptadien-2-one	-	-	-	-	989063	80114
358	Undecanal	1037587	85082	-	-	-	-
359	1,1-Diethoxydecane	583359	48419	394313	32728	-	-
360	2-Methyl-Benzaldehyde	-	-	-	-	582697	48947
361	3-Methyl-Benzaldehyde	494499	42032	196231	16680	1122432	95407
362	4-Methyl-Benzaldehyde	102755	8837	78652	6764	-	-
363	(E)-2-Decenal	-	-	49459	2572	-	-
364	(Z)-2-Decenal	1724434	91395	-	-	726162	38487
365	Phenylethanal	34409478	1858112	36237395	1956819	49808940	2689683
366	Acetophenone	14801364	814075	27343283	1503881	54879434	3018369
367	Cryptone	-	-	-	-	271168	15185
368	6,10-Dimethyl-2-undecanone	1474840	84066	-	-	-	-
369	Carvotanacetone	2398203	139096	609463	35349	5743850	333143
370	2-Hydroxy-benzaldehyde	10978709	647744	603877	35629	1919386	113244
371	(E,E)-2,4-Nonadienal	1404433	84266	285180	17111	1978499	118710

372	Dodecanal	546078	33311	-	-	-	-
373	(2,2-Diethoxyethyl)-benzene	25083741	1555192	24337649	1508934	51568845	3197268
374	4-Ethyl-benzaldehyde	314236	19797	-	-	672073	42341
375	p-Menth-1-en-7-al	118146	7561	-	-	-	-
376	1-Phenyl-1-propanone	2111485	137247	1288759	83769	7198327	467891
377	p-Menth-1-en-3-one	215837	14245	-	-	835548	55146
378	4-(1-Methylethyl)-benzaldehyde	504956	33832	138060	9250	-	-
379	1-(4-Methylphenyl)-ethanone	-	-	912243	62033	-	-
380	1-Phenyl-1-butanone	2079272	143470	578309	39903	5088600	351113
381	4-Phenyl-2-butanone	87718	6140	107397	7518	206603	14462
382	5-(2-Propenyl)-1,3-benzodioxole	-	-	442960	31450	3100679	220148
383	4-Hydroxy-2-methylacetophenone	-	-	-	-	400952	28869
384	2-Phenyl-2-butenal	2307212	168426	5339681	389797	7661878	559317
385	4-Methyl-2-phenyl-2-pentenal	164020	12137	-	-	473812	35062
386	Maltol	-	-	-	-	124309	9323
387	2-Pentadecanone	4365253	331759	-	-	-	-
388	5-Methyl-2-phenyl-2-hexenal	243353	18738	-	-	368530	28377
389	6,10,14-Trimethyl-2-pentadecanone	-	-	281093	21925	1196916	93359
390	1-(4-Methoxyphenyl)-ethanone	78180	6176	-	-	1115619	88134
391	1-(3-Methoxyphenyl)-ethanone	-	-	758048	60644	-	-
392	1-(4-Methoxyphenyl)-2-propanone	861667	69795	-	-	1241050	100525

393	4-Methoxy-6-(2-propenyl)-1,3-benzodioxole	-	-	15014	1231	-	-
394	1-(2-Hydroxy-4-methoxyphenyl)-ethanone	1114047	92466	7130673	591846	35758106	2967923
	**Furans**						
395	Furan	172934	14699	-	-	-	-
396	2-Methyl-furan	436832	37568	-	-	321009	27607
397	2-Ethyl-furan	1059254	92155	-	-	-	-
398	Dihydro-2-methyl-3(2H)-furanone	-	-	150296	13226	-	-
399	3-Furaldehyde	-	-	174983	15573	38084	3389
400	2-Furaldehyde diethyl acetal	3555099	319959	-	-	-	-
401	2-Furancarboxaldehyde	364666	33185	391630	35638	1622244	147624
402	Benzofuran	-	-	1505419	67744	3012525	135564
403	1-(2-Furanyl)-ethanone	8607724	395955	40218887	1850069	161782	7442
404	Tetrahydro-2-furanmethanol	547159	25716	-	-	-	-
405	1-(2-Furanyl)-1-propanone	-	-	12379787	594230	1619844	77753
406	5-Methyl-furfural	10612803	520027	149297	7316	63093	3092
407	Furfuryl propionate	-	-	-	-	759547	37977
408	2-Methyl-benzofuran	1188841	60631	-	-	-	-
409	2-Furfurylfuran	896111	46598	-	-	671878	34938
410	2-Acetyl-5-methylfuran	421734	22352	82894	4393	-	-
411	Ethyl 2-furoate	17686773	955086	23094921	1247126	59239552	3198936
412	5-Ethyl-2-furaldehyde	109327	6013	238626	13124	227458	12510

413	2-Furanmethanol	1506672	84374	-	-	623303	34905
414	2-Butanoylfuran	-	-	199444	11368	3703913	211123
415	Furfuryl butyrate	3273188	189845	605070	35094	728212	42236
416	2-Furfuryl-5-methylfuran	222510	13128	-	-	-	-
417	5-Methyl-2-propionylfuran	-	-	449847	26991	851433	51086
418	Propyl 2-furoate	910682	55552	262400	16006	2450323	149470
419	Furfuryl pentanoate	105224	6524	47345	2935	90878	5634
420	Furfuryl hexanoate	22084209	1391305	3719716	234342	12013859	756873
421	2-Hexanoylfuran	-	-	-	-	840142	53769
422	Dibenzofuran	2293934	149106	543148	35305	167174	10866
423	5-Hydroxymethylfurfural	-	-	364833	24079	74330	4906
	**Nitrogen compounds**						
424	Pyridine	-	-	423182	28776	994460	67623
425	Trimethyl-oxazole	-	-	406865	28074	-	-
426	Pyrazine	-	-	-	-	208214	14575
427	2-Methyl pyrazine	-	-	83202	5907	-	-
428	2,5-Dimethyl-pyrazine	-	-	702853	50605	3367077	242430
429	2,6-Dimethyl-pyrazine	-	-	1913551	139689	-	-
430	Ethyl-pyrazine	-	-	267924	19826	-	-
431	2,3-Dimethyl-pyrazine	-	-	1242690	93202	625362	46902
432	Isopropylpyrazine	-	-	120792	9180	98578	7492

433	2-Ethyl-6-methyl-pyrazine	-	-	1705490	131323	258352	19893
434	2-Methyl-3-isopropylpyrazine	-	-	87029	6788	-	-
435	2-Ethyl-5-methyl-pyrazine	-	-	274812	21710	-	-
436	Trimethyl-pyrazine	846909	67753	8704998	696400	9491474	759318
437	2,6-Diethyl-pyrazine	-	-	-	-	235092	21158
438	3-Ethyl-2,5-dimethyl-pyrazine	139166	13917	562146	56215	738285	73829
439	2,3-Dimethyl-5-ethylpyrazine	-	-	1777799	195558	2003270	220360
440	Tetramethyl-pyrazine	3234143	194049	25382464	1522948	17916862	1075012
441	3,5-Diethyl-2-methyl-pyrazine	-	-	-	-	183053	11349
442	2,3,5-Trimethyl-6-ethylpyrazine	-	-	-	-	1164356	74519
443	1H-Pyrrole	380312	25101	859338	56716	594218	39218
444	2-Methyl-1H-pyrrole	-	-	-	-	360582	24520
445	3-Methyl-1H-pyrrole	-	-	94848	6639	893985	62579
446	2-Pentyl-Pyridine	-	-	212086	15270	-	-
447	2-Formyl-1-methylpyrrole	-	-	152168	11260	-	-
448	2-Acetyl-6-methylpyridine	-	-	358221	27225	-	-
449	2,3-Dimethyl-5-isopentylpyrazine	-	-	-	-	87481	6824
450	Quinoline	-	-	49214	3937	-	-
451	2-Acetylpyrrole	116000	9512	1708061	140061	203061	16651
452	2-Formylpyrrole	100422	8435	787607	66159	-	-
	**Sulfur compounds**						

453	Methanethiol	17980056	1582245	-	-	704765	62019
454	Dimethyl sulfide	353186	25076	-	-	-	-
455	Methyl thiolacetate	509075	36653	674068	48533	-	-
456	Dimethyl disulfide	185282279	13525606	92806996	6774911	40528354	2958570
457	S-Methyl propanethioate	149016	11027	469766	34763	452243	33466
458	Methyl ethyl disulfide	-	-	228216	17116	-	-
459	S-Methyl ester butanethioic acid	21100363	1603628	29268184	2224382	8011206	608852
460	Thiazole	-	-	233446	17975	387213	29815
461	Dimethyl trisulphide	69373	5411	29336074	2288214	8989421	701175
462	S-Methyl hexanethioate	34583030	2732059	3277599	258930	251289	19852
463	Methyl pentyl disulfide	-	-	-	-	331673	26534
464	4,5-Dimethyl-2-isopropyl-thiazole	-	-	-	-	79667	6453
465	Ethyl 2-(methylthio)acetate	154790	12693	-	-	-	-
466	Methional	1748093	145092	1030464	85529	1675740	139086
467	2-Pentyl-thiophene	183530	15417	127659	10723	385221	32359
468	Furfuryl methyl sulfide	1329630	113019	-	-	1892206	160838
469	4,5-Dimethyl-2-isobutylthiazole	-	-	-	-	279987	24079
470	2-(Methylthio)ethanol	32270	1678	-	-	-	-
471	Methyl propyl trisulfide	1395732	73974	-	-	-	-
472	Ethyl 3-(methylthio)propionate	7258259	391946	4144047	223779	30640434	1654583
473	2,5-Dimethyl-1,3,4-trithiolane	223811	12310	-	-	303046	16668

474	3-(Methylthio)propyl acetate	118146	6616	-	-	-	-
475	2,4,5-Trithiahexane	5489595	312907	1093456	62327	12531933	714320
476	Methyl benzyl sulfide	192529	11167	-	-	282660	16394
477	3-Thiophenecarboxaldehyde	305628	18032	283932	16752	309785	18277
478	2-Thiophenecarboxaldehyde	212586147	12755169	3957333	237440	1131135359	67868122
479	Methionol	162384	9905	150563	9184	108786	6636
480	5-Methyl-2-formylthiophene	204230	12662	87681	5436	759939	47116
481	Dimethyl tetrasulphide	-	-	-	-	57620	3630
482	1,2,4-Trithiolane	246095	15750	-	-	41966	2686
483	3-Acetylthiophene	-	-	139269	9052	-	-
484	2-Acetylthiophen	-	-	-	-	459773	30345
485	Furfuryl methyl disulfide	1833204	122825	1788668	119841	18687219	1252044
486	3-Methyl-2-thiophenecarbaldehyde	8371934	569292	540941	36784	5341662	363233
487	1-(2-Thienyl)propanone	75104	5182	-	-	-	-
488	Benzothiazole	204159	14291	1184171	82892	764993	53550
489	2-Phenylthiophene	104707	7434	-	-	-	-
	**Phenols**						
490	2-Methoxy phenol	24190543	1765910	1626545	118738	9636718	703480
491	Di-ter-butyl p-cresol	10933508	809080	2467790	182616	1688443	124945
492	2-Methoxy-4-methylphenol	20011936	1500895	74285744	5571431	74499491	5587462
493	Phenol	112323	8537	39826797	3026837	199061	15129

494	2-Methylphenol	1566609	120629	3671169	282680	3445312	265289
495	2-Methoxy-4-ethylphenol	9742421	759909	23854184	1860626	57350333	4473326
496	p-Cresol	66137	5225	85926604	6788202	51503723	4068794
497	2-Methoxy-4-propylphenol	49459	3957	311353	24908	-	-
498	3-Ethylphenol	-	-	-	-	772484	62571
499	2-tert-Butylphenol	-	-	-	-	38722	3175
500	4-Ethylphenol	8129380	674739	23603547	1959094	46984070	3899678
501	5-Methyl-2-isopropylphenol	426916	35861	1523874	128005	1434914	120533
502	2-Methoxy-4-vinylphenol	-	-	751023	63837	-	-
503	2-Methyl-5-isopropylphenol	100459	8639	-	-	-	-
504	4-Propylphenol	238850	20780	308508	26840	2272521	197709
505	2,6-Dimethoxyphenol	-	-	70467	6201	64359	5664
506	2,4-Di-tert-butylphenol	426179	37930	1540199	137078	925567	82375
507	4-Ethenylphenol	-	-	-	-	170578	15352
	**Terpenes**						
508	delta-3-Carene	-	-	275084	12379	-	-
509	α-Limonene	505494	23253	241636	11115	-	-
510	1,8-Cineole	6930888	325752	582863	27395	3883944	182545
511	Terpinolene	98328	4720	-	-	-	-
512	α-Thujone	-	-	-	-	33943	1663
513	trans-Linalool oxide	-	-	-	-	703090	35155

514	cis-Linalool oxide	2417631	123299	2134672	108868	6046992	308397
515	α-Longipinene	104594	5439	-	-	-	-
516	α-Copaene	-	-	-	-	456023	24169
517	Daucene	267678	14455	-	-	-	-
518	Longicyclene	3147074	173089	-	-	-	-
519	Theaspirane B	18610	1042	-	-	-	-
520	Camphor	5049195	287804	1918995	109383	14350772	817994
521	(-)-Camphor	11903953	690429	-	-	-	-
522	Vitispirane	1255186	74056	-	-	184995	10915
523	α-Gurjunene	5041306	302478	-	-	219694	13182
524	Linalool	7344560	448018	1217089	74242	7146698	435949
525	Theaspirane	151856	9415	-	-	-	-
526	α-Cedrene	-	-	1087599	68519	-	-
527	Carvomenthone	1121930	71804	-	-	337650	21610
528	β-Funebrene	-	-	107854	7011	188784	12271
529	Junipene	14105904	930990	-	-	-	-
530	D-Fenchyl alcohol	1029951	69007	-	-	1102870	73892
531	α-trans-Bergamotene	573526	39000	-	-	-	-
532	α-Guaiene	1395732	96306	-	-	-	-
533	β-Elemene	2009887	140692	-	-	-	-
534	Calarene	30536066	2168061	1157650	82193	174785	12410

535	trans-Caryophyllene	276168	19884	-	-	264420	19038
536	Terpinen-4-ol	4403323	321443	319168	23299	8098403	591183
537	Isophorone	1558470	115327	56097	4151	4874869	360740
538	trans-Edulan	-	-	-	-	202070	15155
539	β-Terpineol	180198	13695	-	-	-	-
540	β-Cyclocitral	460377	35449	-	-	508713	39171
541	α-Patchoulene	754174	58826	-	-	-	-
542	Alloaromadendrene	3791114	299498	-	-	168711	13328
543	β-Barbatene	444598	35568	-	-	-	-
544	γ-Gurjunene	1566571	140991	-	-	-	-
545	Isoborneol	71798	7180	-	-	-	-
546	α-Humulene	137976	15177	-	-	246928	27162
547	L-Borneol	1033708	62022	-	-	-	-
548	α-Terpineol	5374459	333216	2556510	158504	-	-
549	γ-Amorphene	4660098	298246	-	-	-	-
550	Ledene	1284581	84782	-	-	-	-
551	trans-Borneol	103216	7019	2403930	163467	2237130	152125
552	β-Chamigrene	709932	49695	-	-	-	-
553	Valencene	452235	32561	-	-	-	-
554	α-bisabolene	3580062	264925	30207	2235	240100	17767
555	Germacrene A	-	-	-	-	412245	31331

556	α-Chamigrene	50571	3945	-	-	413689	32268
557	δ-Cadinene	14210759	1136861	888095	71048	1008431	80674
558	β-Citronellol	432682	35480	-	-	-	-
559	7 epi-a-Selinene	228452	19190	-	-	-	-
560	α-Curcumene	1816428	156213	103639	8913	-	-
561	Nerol	-	-	-	-	121972	10734
562	Isogeraniol	-	-	-	-	48526	3445
563	β-Damascenone	3077793	221601	-	-	547245	39402
564	Dihydro-β-ionone	476858	34811	92771	6772	184811	13491
565	L-calamenene	58939	4361	76061	5629	43873	3247
566	Geraniol	552207	41416	-	-	699664	52475
567	trans-Geranylacetone	12870057	978124	3980433	302513	-	-
568	Geosmin	932703	71818	563398	43382	67246	5178
569	α-Ionone	186518	14548	-	-	-	-
570	α-Dehydro-himachalene	125865	9943	-	-	-	-
571	α-Calacorene	113310	9065	55107	4409	53888	4311
572	Palustrol	691765	56033	209429	16964	212867	17242
573	trans-β-Ionone	278310	22821	80845	6629	447927	36730
574	cis-Jasmone	-	-	-	-	45823	3803
575	β-Caryophyllene oxide	109293	9181	143285	12036	21429	1800
576	D-Nerolidol	348336	29609	-	-	400307	34026

577	E-Nerolidol	-	-	422257	36314	-	-
578	Epicubenol	113310	5892	-	-	-	-
579	α-Corocalene	84119	4458	-	-	-	-
580	Cubenol	-	-	95976	5183	-	-
581	6-Isocedrol	167743	9226	-	-	-	-
582	α-Cedrol	-	-	1018100	57014	1263118	70735
583	β-Bisabolol	-	-	-	-	119517	6812
584	Torreyol	-	-	496264	28783	764401	44335
585	α-Cadinol	634215	37419	-	-	966611	57030
586	α-Eudesmol	-	-	-	-	133851	8031
587	β-Eudesmol	-	-	-	-	1133257	69129
588	Farnesol	-	-	54435	3375	-	-
589	9H-Fluorene	-	-	260964	16441	703901	44346
	**Lactones**						
590	γ-Pentalactone	1944867	126416	-	-	61953	4027
591	Butyrolactone	1923674	126962	710199	46873	498097	32874
592	γ-Vinyl-γ-valerolactone	-	-	607048	40672	-	-
593	γ-Caprolactone	29462109	2003423	-	-	-	-
594	α-Methyl-γ-crotonolactone	-	-	267712	18472	7637688	527000
595	3,4-Dimethyl-2,5-furandione	-	-	131962	9237	-	-
596	γ-Crotonolactone	-	-	1374282	97574	2203820	156471

597	δ-Hexalactone	651278	46892	1363211	98151	753277	54236
598	γ-Octalactone	-	-	84613	6177	3953827	288629
599	δ-Octalactone	-	-	118699	8784	433161	32054
600	γ-Nonalactone	8263612	619771	-	-	-	-
601	γ-Carboethoxy-γ-butyrolactone	-	-	-	-	89450	6798
602	γ-Decalactone	1101223	84794	122264	9414	8383106	645499
603	δ-Decalactone	-	-	25976	2026	1057780	82507
604	γ-Dodecalactone	-	-	58387	4613	-	-
605	γ-6-(Z)-Dodecenolactone	342131	27370	1534696	122776	9342063	747365
606	δ-Heptyl-δ-valerolactone	-	-	-	-	681955	55238

[a]: Peak area of each compound from different extract method.
